# Multicomponent approach reveals differences in affective responses among children and adolescents

**DOI:** 10.1038/s41598-025-94309-2

**Published:** 2025-03-25

**Authors:** Kseniia Konopkina, Hilla Hirvaskoski, Jari K. Hietanen, Heini Saarimäki

**Affiliations:** 1 Human Information Processing Laboratory, Faculty of Social Sciences, Tampere University, Tampere, FI-33014 Finland; 2 Department of Psychology, University of Otago, Dunedin, 9016 New Zealand

**Keywords:** Naturalistic stimuli, Emotional development, Psychophysiology, Self-reports, Human behaviour, Physiology, Psychology

## Abstract

Investigating age-related shifts in affective responses to emotionally salient stimuli is key to comprehending emotional development during childhood and adolescence. Most of the research regarding emotional experiences has focused on adults, while the understanding of the development of emotional experiences across childhood remains elusive. To address this gap, we explored whether physiological and behavioural responses as well as self-reported emotions elicited in children and adolescents by naturalistic stimuli differ from those in adults. We developed a set of emotional videos to elicit different emotions – fear, joy, anger, sadness, amusement, and tenderness - and measured emotional intensity ratings, electrocardiography, and eye movements from 8–15-year-old children and adults during the viewing of the videos. We identified age-related changes in all measured responses. Emotional intensity and behavioural responses varied across emotion categories. Furthermore, specific emotions showed different maturation patterns. The study highlights the importance of a multicomponent approach to accurately discern and understand emotional states.

**Supplementary Information:**

The online version contains supplementary material available at 10.1038/s41598-025-94309-2.

## Introduction

Emotions are complex mental states that involve several components, including subjective experiences, behavioural responses, and physiological changes^1^. The component process model posits that categorical emotions, such as fear or joy, arise from integrating these components^2^. While much research has focused on these components in adults (for reviews, see, e.g^3,4^), gaps in understanding remain, particularly in how these components interact across development.

Subjective experiences, commonly referred to as feelings, reflect the conscious aspects of emotions^5,6^. These are often assessed through self-report ratings of some aspect of emotional experience, such as the intensity of discrete emotions^7–9^, or valence and arousal^10,11^. However, the reliability of self-reports in young children remains contentious. Children as young as three years of age can provide self-reports of their emotions, which are aligned with their vocal, bodily, and facial behaviours^12,13^. Self-reports of bodily changes associated with basic emotions are already distinct in six-year-old children and become increasingly similar to those reported by adults during maturation^14^. Nevertheless, young preschool children tend to provide extreme responses when using Likert-type scales to rate their emotional states^15^ and rate the intensity higher compared to external coders^13^. Additionally, children may lack emotional vocabulary or awareness of their emotional states^16^. A more comprehensive understanding of children’s emotional states may be gained by using self-reports and behavioural measures in combination with psychophysiological measures^16–18^.

Emotional behaviours can be manifested in facial movements^19^, body posture and movements^20^, vocal prosody^21^, blink rates^22^, or other gaze behaviours^23^. For instance, Maffei and Angrilli^24^ found that blink rates decrease during compassionate and scenic scenes and increase during fear-inducing clips. Similarly, neural networks can infer emotional states from blink data, with the highest accuracy observed during fear-inducing clips^25^. Significant differences in blink modulation were found in children when they viewed unpleasant pictures compared to neutral and pleasant ones^26,27^, potentially due to the high arousal of unpleasant stimuli (see, e.g^28^).

Physiological indicators of emotional states include heart rate^29,30^, respiration^31,32^, skin conductance^33^, muscle tension^34^, and hormone levels^35^. While adult emotional physiology is well-documented (for meta-analyses, see^36,37^), fewer studies focus on children. Sacrey et al.^38^ reviewed studies using cardiac activity to measure physiological responses to emotions in children from newborns to 4-year-olds and found similar patterns to adults, such as increased HR and decreased HRV in fear, anger, or anxiety (for a meta-analysis, see^36^). Several studies^26,39,40^ have demonstrated that school-aged children display comparable but weaker physiological responses to emotional stimuli than adults. In general, cardiac measures are considered reliable for assessing emotional states in children^18^. However, when assessing age differences in physiological responses to affective stimuli, developmental changes in cardiac functioning must be considered^41^.

These three components of emotions – subjective experiences, behavioural responses, and physiological changes – are interconnected^42,43^. However, research on the development of children’s emotions has often focused on isolated single components^44–46^. While some studies have explored combinations of two components^13,47,48^, studies involving a multicomponent approach with more than two components are rare. Notably, Leventon et al.^40^ combined self-reports, physiological measures (heart rate, heart rate variability, and event-related potentials), and behavioural memory performance, while McManis et al.^26^ assessed physiological responses (heart rate), self-report, and behavioural responses (EMG and blinks). Both studies used affective pictures to induce emotional states, although recent research suggests that dynamic, naturalistic stimuli are more effective in eliciting emotional responses^49,50^, thus increasing the ecological validity^51^.

This study investigated age-related changes in affective responses to emotionally salient stimuli, aiming to determine the extent of alignment between children’s and adults’ emotional reactions. This research addresses a gap in the current understanding of emotional development during middle childhood and adolescence, as most existing studies have primarily focused on adults and often limited their scope to just one or two emotional components. In contrast, our study employed a multicomponent approach, examining three modalities of emotional reactions – self-reported, physiological, and behavioural responses – in 8–15-year-olds compared to those in adults when exposed to emotional videos. Given the inclusion of less-studied positive emotions and the novel application of a multimodal approach, we adopted an exploratory framework without placing specific hypotheses. We used linear mixed-effects models (LMMs) to examine whether emotional responses associated with different emotion categories vary across ages and representational similarity analysis (RSA) to investigate whether specific emotion categories are associated with distinct physiological and behavioural response patterns and how these similarities emerge across development. Our analytical approach is guided by the component process model of emotions^43,52^, which suggests that emotional responses consist of distinct yet interdependent components that collectively shape the final subjective experience or “feeling.” In this framework, early components, such as physiological and behavioural responses, contribute to the eventual categorisation and labelling of emotions.

## Method

### Participants

The sample comprised children and adolescents aged 8 to 15 years old (*N* = 90, 48 girls, 42 boys, mean age = 11 years, SD = 2.15) and a reference group of young adults (*N* = 30, 15 women, 14 men, 1 non-binary, age range 19–29 years, mean age = 22.2 years, SD = 2.5). All participants were Caucasian and spoke Finnish as their native language. The data were collected in the facilities of the Human Information Processing Laboratory at Tampere University. Participants were recruited through online advertisements on social media platforms and mailing lists. Those interested, along with their guardians, registered via an online form and were later invited to the lab for participation. Young adults were recruited through a research methodology course at Tampere University. Upon registration, all participants or their guardians filled out self-reports of background variables, including gender, age, and native language. Children and adolescents received cinema tickets as compensation for their participation, whereas young adults were provided with course credits.

Participation in the study was voluntary, and participants had the option to discontinue or withdraw from the study at any point. Prior to participation, participants and their caregivers were provided with information about the study and given the opportunity to ask questions. Informed consent was obtained, with caregivers consenting on behalf of children aged 8 to 14. For adolescents aged 15, their own consent was sufficient, although they were encouraged to discuss their participation with their caregivers. The sample is relatively homogeneous, particularly in terms of socio-economic status, race, and ethnicity, due to their low variation in the Tampere region where data were collected. At the time of data collection, over 90% of the population of the Tampere region were native Finns and spoke Finnish as their native language. Race, ethnicity, and socioeconomic status were not explicitly collected from our sample. Ethical statement for the experiment was obtained from the Ethics Committee of the Tampere Region. All procedures were conducted in accordance with the relevant guidelines and regulations outlined in the Declaration of Helsinki.

### Video task

The study consisted of a single 2-hour-long laboratory visit. The video task was one of several measurements included in the study and was conducted after the participant filled out self-report questionnaires and completed a facial expression recognition task, an emotion recognition task, and an interview of emotional experiences. Only the video task data were analysed in this study. The video task lasted for less than an hour: the total duration of videos was 33 min and 19 s, with self-paced breaks for filling out self-reports after each video. Participants were allowed to take breaks between videos when necessary.

In the video task, participants watched 42 brief, approximately one-minute-long video clips depicting a range of emotional situations (duration range = [0:18 − 1:10 min.]; total duration = 33:19 min.). To select the video stimuli for this study, we first identified 135 scenes portraying emotional content from Finnish-language children’s films and TV series and conducted a pre-evaluation survey where 14 adult participants (2 males, mean age = 23.6, SD = 3.1, range 21–33 years) were asked to freely label the emotion category experienced during each clip (Supplementary Table 1). All selected movies and TV series for this study adhered to the Finniah age classification system, being rated either “S” (suitable for all ages) or “7” (appropriate for viewers aged 7 and older). Following the pre-evaluation survey, a final set of 42 videos was chosen to represent six target emotion categories (seven clips per each category; see Supplementary Table 2 for content and familiarity of the clips): joy, anger, sadness, fear, tenderness, and amusement. All video stimuli are available from the corresponding author on request.

Our selection of emotion categories was guided by both dimensional and discrete models of affect. While dimensional models categorise emotions based on valence and arousal^53,54^, discrete emotion theories emphasise distinct, biologically-rooted emotion categories^55,56^. Recent perspectives argue that these models are not mutually exclusive^57–59^. Historically, emotion research has focused largely on the taxonomy of six basic emotions^36,60^, which included only one positive emotion, joy, based on the belief that it was the only positive state with a universally recognised expression. However, more recent findings^61–63^ have identified a broader range of positive affective states that are expressed and recognised across cultures. Given the limited focus on positive emotions in past research, our study aims to balance positive and negative emotions and capture a range of emotional intensities from subtle to intense^64–66^. Therefore, we included tenderness as a low-arousal positive emotion, and joy and amusement as high-arousal positive emotions. Correspondingly, we selected sadness as a low-arousal negative emotion, with fear and anger as high-arousal negative emotions. While more emotions could have been included, we selected a representative subset that balanced valence and arousal while maintaining a feasible experiment length.

The 42 video clips were organised into six separate runs, with each run comprising seven videos. Each run contained one or two video clips from each of the six emotion categories. Both the ordering of the runs and the sequence of videos within them were randomised for each participant. During each run, participants viewed one video clip at a time, with a pause after the stimulus presentation to allow them to rate the emotions they experienced during the clip. The video stimuli were presented on a 23-inch computer screen and the stimulus delivery was controlled with in-house developed software built with the Unity Experiment Framework (UXF). Audio was delivered through loudspeakers with a constant sound level for all participants.

We encouraged participants to take breaks between runs, with at least one break being mandatory during the experiment. Participants were advised to drink a beverage or simply stretch their muscles during these breaks. To maintain consistency and to support the participants, a research assistant was present in the laboratory throughout the study. Quality of the ECG signal was visually inspected throughout the experiment, and participants were encouraged to sit still or take breaks when motion was detected. The video task was successfully completed by 117 participants: three participants interrupted the video task after 4 or 5 runs (one 9-year-old, one 10-year-old, one 15-year-old). The data collected before interrupting the experiment were included in the analyses.

### Ratings

The rating procedure aimed to quantitatively capture participants’ emotional experiences during the video task. Following the viewing of each video clip, participants provided their emotional ratings using a tablet. The emotional rating interface was implemented with the Gorilla Experiment Builder (www.gorilla.sc;^67^). The rating interface employed a slider, which enabled participants to indicate the intensity of their experienced emotion for six emotion categories, including three positive emotions (joy, tenderness, amusement) and three negative emotions (anger, fear, sadness). Text labels of emotion categories were complemented with corresponding emojis (Supplementary Fig. 1). Before the video task, children participated in an interview of emotional experiences, where they were given each emotion category and asked to describe a recent situation where they had felt this emotion. This interview task was used to confirm that all participants understood all emotion categories in this study. If the participant was not familiar with the emotion category, they were given a standardised description to ensure understanding of all emotion labels.

Participants were instructed to use the slider interface to rate the intensity of their emotional experience. In cases where a participant did not experience a specific emotion, they were instructed to leave the slider at its default position on the left end (no emotion at all). Conversely, moving the slider towards the right end of the scale indicated a high level of emotional intensity for the corresponding emotion. As a result, we obtained rating data ranging from 0 (no emotion at all) to 100 (maximum intensity). Participants practiced using the rating interface before the video task.

We collected rating data successfully for all but one participant (one 13-year-old: all data missing due to a technical error in the rating interface). Additionally, some participants had missing ratings for 1–2 clips out of 42 (one missing rating: *N* = 26; two missing ratings: *N* = 9; see Supplementary Table 3 for age distribution of missing data) mostly in the first run of their experiment. These missing data were due to problems with using the rating interface despite the training before.

### Eye movements

The behavioural response to emotional clips was calculated as the percentage of time when participants’ gaze was fixated on the screen. During the video task, participants’ eye movements were tracked using a Tobii Pro X3-120 eye tracker attached to the 23-inch monitor that displayed the video stimuli. Participants were positioned approximately 50 cm away from the monitor. Before the video task commenced, a calibration task was performed, and when necessary, it was repeated until a satisfactory calibration result was achieved, with a maximum limit of three calibration attempts. If the calibration limit was reached, the experiment proceeded without eye-tracking data collection, as data for other modalities could still be collected. Eye-tracking data were not collected for five participants (three adults, one 11-year-old, one 13-year-old) due to calibration problems persisting after three calibration attempts. During the calibration process, participants were instructed to fixate their gaze on a dot that appeared at various locations on the screen. To ensure participants’ comfort and to allow them maintaining a natural posture, no headrests or chinrests were used during the video viewing sessions. Participants were encouraged to avoid excessive movement during the videos.

Eye-tracking log files recorded timestamps and gaze coordinates on the X and Y axes. Instances of undetected gaze, resulting from blinks or gaze shifts beyond the screen, were marked by coordinates (-1920, -1080). For each participant and video clip, the total gaze duration was calculated by summing periods when gaze was detected. To adjust for variations in video clip length, this duration was represented as a percentage of the total clip duration.

### Heart rate

Physiological responses during video viewing were measured with heart rate metrics calculated from the electrocardiogram (ECG) data. ECG data were collected using two self-adhesive electrodes, placed on the lower rib and clavicle, and Net Station 4.5.1 software (Electrical Geodesics, Inc.) at a sampling rate of 250 Hz. ECG data were not collected for six participants (one 8-year-old, one 10-year-old, one 12-year-old, two 13-year-olds, one 15-year-old) due to technical problems.

ECG data were processed using Python (version 3.8.13), employing standard libraries for data manipulation (pandas 1.4.1), general computing (numpy 1.21.5), and graphical representation (matplotlib 3.5.1, seaborn 0.11.2). The heart rate time series from the comprehensive recording was divided into 42 segments, corresponding to individual video clips. Raw ECG data preprocessing, peak detection, and peak correction were automated through the open-source Neurokit2 (version 0.1.7) library in Python^68^. In cases of substantial noise and motion artifacts, three distinct peak detection algorithms (‘neurokit’, ‘kalidas2017’, ‘promac’) were applied, with the most suitable method selected for each instance via visual inspection. Following this, data were examined and corrected manually to verify accurate peak identification. Segments with unidentifiable peaks were excluded from further analysis. Altogether, we excluded segments from seven participants (between one and 13 segments per participant). The remaining intact segments were used for calculating the average responses for each emotion category.

After peak detection in each video clip, we calculated the average heart rate (HR) in beats per minute (BPM). To quantify heart rate variability (HRV), we employed the RMSSD method, which represents the root mean square of successive differences of the RR intervals. RMSSD specifically quantifies the short-term variability in heart rate and is particularly sensitive to changes in the parasympathetic nervous system activity^69^.

Finally, to investigate the effect of motion in our data, we quantified motion from ECG raw signal. We calculated the number of trials during which the automatic peak detection failed and peaks needed to be manually corrected. To estimate motion, we calculated the percentage of trials per participants where manual correction was required, as the noisy signal was related motion artefacts. We then calculated correlations between motion and other variables. We found no correlation between motion, gaze to screen, BPM, and RMSSD, suggesting that motion did not fully explain our findings. We also calculated correlation between age and motion. While there was a significant correlation between age and motion in the whole sample (*r*=-.40, *p* < .001), when calculating correlation in children only, we found no correlation (*r* = .04, *p* = .733).

### Statistical analyses

#### Intensity heatmaps of experienced emotions

To evaluate the effectiveness of video clips in inducing the intended emotional responses, we employed descriptive analyses. We used heatmaps to visually represent the intensity of self-reported emotions for each clip, averaged across age groups. Participants were grouped by age as follows: young children 8–9 (*N* = 29), older children 10–11 (*N* = 25), young teenagers 12–13 (*N* = 23), older teenagers 14–15 (*N* = 13), and adults (*N* = 30, mean age = 22.2, SD = 2.5, range 19–29). These heatmaps, ranging from 0 (no intensity) to 100 (maximum intensity), allowed us to observe the correspondence between the intended and reported emotions. Heatmaps were generated using Python (version 3.9.18), utilising the *numpy* and *pandas* packages for data manipulation, and *seaborn* for data visualisation.

#### Linear mixed-effects models

We employed three distinct linear mixed-effects models (LMMs) using the *lme4* package (version 4.1.3) in the statistical software R (version 4.1.2) to assess the effects of age and emotion category of clips on emotional responses: experienced intensity ratings, physiological responses (HR, HRV), and percentage of gaze on screen. To handle the categorical variable representing emotion categories, we adopted orthogonal sum-to-zero contrast coding. In this coding approach, we set the reference level “Amusement” to − 1, which allowed us to measure deviations from the grand mean and also assess interaction effects by examining deviations from the expected values. These models are outlined as follows:

1. Model 1 (Linear age model): In this model, age was treated as a continuous variable, assuming a linear relationship with emotional response. The model was specified as emotion_response ~ age_continuous * emotion_category + (1|id).
2. Model 2 (Polynomial age model): Here, age was included as a polynomial continuous variable to capture potential non-linear effects. The model was specified as emotion_response ~ (age_continuous + age_continuous^2) * emotion_category + (1|id).
3. Model 3 (Categorical age model): Age was treated as a categorical variable, with distinct age groups compared to adults (reference group). The model was specified as emotion_response ~ age_category * emotion_category + (1|id).

Our analytical approach initially involved a comparative assessment of Model 1, which treats age as a linear continuous variable, and Model 2, incorporating age as a polynomial continuous variable. This comparison relied on the Akaike Information Criterion (AIC) and Bayesian Information Criterion (BIC) for selecting either a linear or non-linear model as more appropriate. We then applied analysis of variance (ANOVA) to test the significance of each fixed effect (age, emotion category, and their interaction) in explaining the variability in the dependent variable (emotional response). In the results section, we report the model that best fits the data, be it linear (Model 1) or polynomial (Model 2). Details regarding the comparison between the models are provided in the supplementary section. Additionally, we conducted post-hoc analyses by making pairwise comparisons and adjusting for multiple comparisons with Tukey’s Honest Significant Difference (HSD) test, to further outline differences between the emotion categories (Supplementary Tables 4–21).

In Model 3, age group and emotion category were considered as categorical predictors, while we included participants as a random effect to account for individual variations. For this analysis, participants were grouped by age in the same manner as in the descriptive analysis, into five groups: 8–9 years old, 10–11 years old, 12–13 years old, 14–15 years old, and adults. To establish a baseline, we used adults as the reference level for the age groups. This model is consistently reported in the results to underline differences compared to the adult group.

#### Representational similarity analysis

To further complement these analyses, Representational Similarity Analysis (RSA) was employed. RSA was selected for its ability to analyse complex multimodal data, capturing subtle, potentially nonlinear patterns in emotional development that may not be evident with traditional methods. While RSA necessitates discretisation of age, this approach complements LMMs, providing a more comprehensive perspective on age-related changes in emotional responses.

We employed Representational Similarity Analysis (RSA;^70^) to analyse age-related variations in the distinctiveness of affective responses to the six target emotion categories. To allow age group comparisons, participants were categorised into the same five age groups as in the linear mixed-effects model analysis above. The analysis focused on physiological and behavioural responses, aiming to discern how distinct these responses are in relation to various emotion categories and to understand the developmental changes they undergo.

For the analysis we used *vegan*, *mosaic*, *pheatmap*, and *lsa* packages in R. First, we normalised HRV, HR, and percentage of gaze on screen within each participant. These were concatenated into vectors so that each participant had a vector of three values for each video clip. Second, individual Representational Dissimilarity Matrices (RDMs) were computed for each participant using Euclidean distance for ratings and cosine distance for the other modalities, capturing the dissimilarity in their emotional responses across stimuli. In the 42 × 42 RDMs, each element represented the dissimilarity between pairs of emotional stimuli for a given participant. Third, these individual matrices were then aggregated within each age group and for each targeted emotion category of clips to produce a group-level, 6 × 6 RDMs, providing an overview of emotional response patterns for each emotion category at the group level. These matrices were used to interpret how different age groups respond to various emotional stimuli, both within and across emotion categories. For reporting purposes, we categorised values below the 10th percentile of the similarity range of each matrix as indicating high similarity, while values between the 10th and 25th percentiles were classified as indicating medium similarity. Guided by the component process model of emotions, we calculated similarity matrices for combined physiological and behavioural responses (HR, HRV, and percentage of gaze on screen) and separately for intensity ratings, representing the final stage of emotional processing. RSA analyses with HR, HRV, and percentage of gaze on screen, considered separately, highlighted the necessity of a multicomponent approach when investigating emotional responses.

Statistical analysis involved comparing the group-level RDMs using the Mantel test, a non-parametric method suitable for assessing the correlation between two distance matrices. The Mantel test between each age group was conducted using Spearman’s rank correlation coefficient, with 5,000 permutations for each comparison to determine statistical significance. The Mantel statistic provides a measure of the similarity between group-level RDMs, while the p-value indicates the statistical significance of this similarity. The resulting p-values determined whether the emotional response patterns were similar across age groups, with a p-value below 0.05 considered indicative of a statistically significant relationship. These results can discern the extent to which emotional responses are consistent or vary across different developmental stages. Finally, we also used Spearman correlation test to evaluate similarity of emotion components.

## Results

### Experienced emotions: intensity heatmaps

**Fig. 1:**
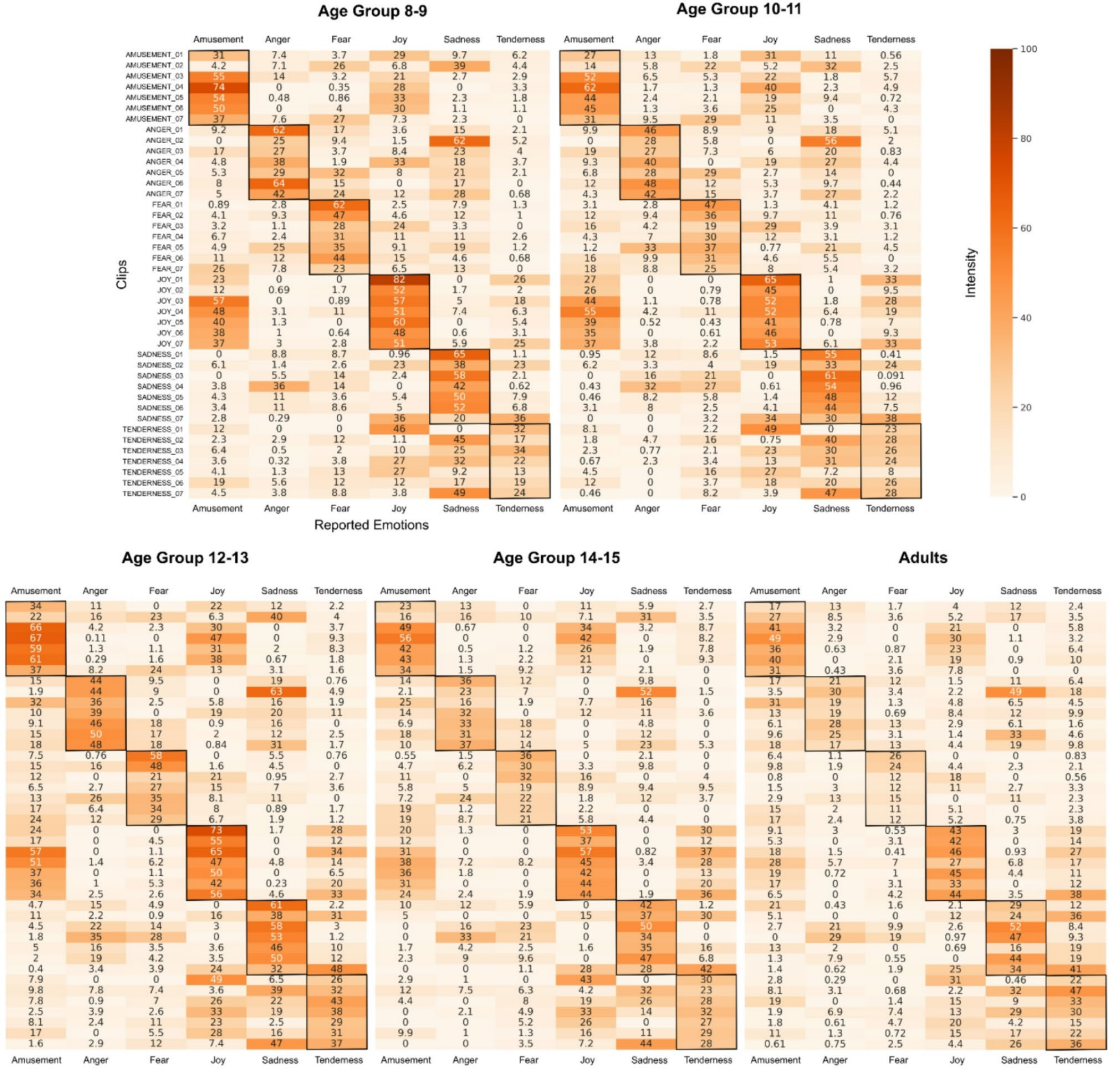
Heatmaps of experienced emotions by age group and clip. The heatmaps display the emotions experienced by participants during each clip, categorised across age groups. Emotion ratings are on a scale from 0 to 100, reflecting the average intensity of emotions experienced within each age group. A darker colour on the heatmap indicates greater emotional intensity. The Y-axis represents the clips (7 clips in each category) along with their intended emotion categories, while the X-axis represents the reported experienced emotions.

First, we evaluated the effectiveness of the video clips in eliciting target emotions. The heatmaps (Fig. 1) show that for most age groups, the pre-selected clips were effective in eliciting the target emotions. Furthermore, the reported intensity of experienced emotions decreased across age.

Target-to-elicited success rates were consistently high for the categories of joy and amusement across most age groups. Moreover, joy and amusement often co-occurred in clips intended to induce either emotion. This co-occurrence was consistent across all age groups, except in adults, where these emotions became more distinct.

Overall, the descriptive analysis of the heatmaps revealed that the clips mostly evoked the intended target emotions, while there was also notable age-related variation in self-reported emotions. For this reason, to complement the original analyses with target emotions, we also conducted the analyses using individualised emotion categories in addition to those based on the predefined target categories. The results demonstrated consistent outcomes across the two approaches; therefore, the following results are reported based on the target emotion analysis. For results of non-target emotions and the individualised emotion analysis, see Supplementary Tables 22–25 and 26–33 in Supplementary Analyses 1 and 2, respectively.

### Linear mixed-effects models

**Fig. 2:**
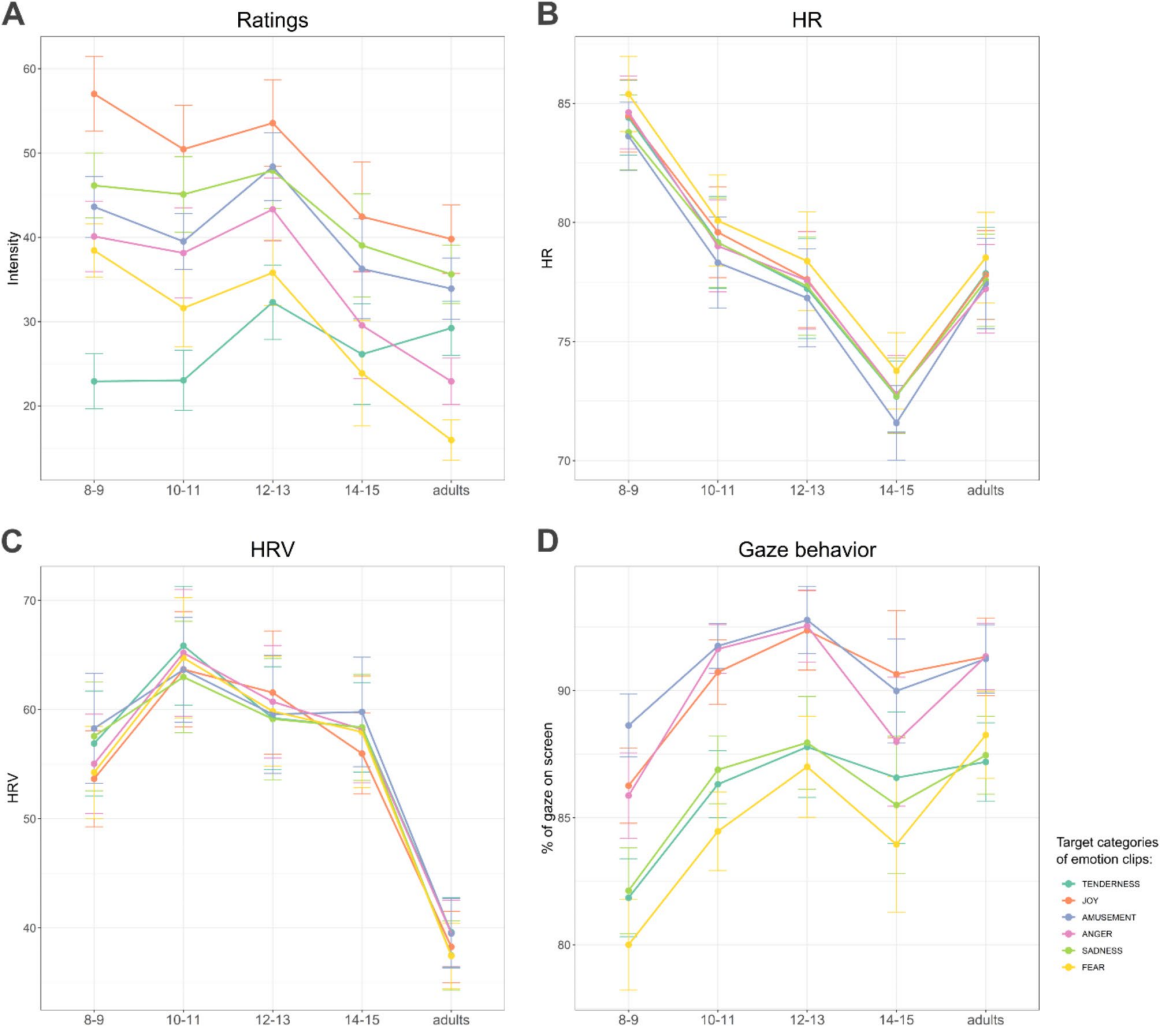
Emotional responses to six emotion categories in each age group. (**A**). Ratings of subjective emotional experience. (**B**). Heart Rate (HR). (**C**). Heart Rate Variability (HRV). (**D**). Percentage of time participants looked on the screen.

### Effects of age and emotion category on self-reported emotional intensity

We first investigated whether children and adults reported similar felt intensity of target emotions and whether there were differences in intensity ratings between emotion categories (Fig. 2A, Supplementary Tables 4–5). The linear model showed main effects of both age (F(1, 116.9) = 10.3, *p* = .002) and emotion category (F(5, 583.9) = 15.3, *p* < .001). In general, self-reported emotional intensity declined with age across all emotion categories (β = -0.86, *p* = .002). We also found a significant interaction effect of age and emotion category (F(5, 583.9) = 7.2, *p* < .001). This interaction was due to increased emotional intensity of tenderness with age (β = 1.20, *p* < .001).

Upon treating age as a categorical variable, children aged 8–9 (β = 11.81, *p* = .004) and 12–13 (β = 13.76, *p* = .002) showed significantly more intense emotional responses relative to adults (Supplementary Table 6). The responses of the 10–11 age group showed (β = 8.4, *p* = .05) a trend towards more intense experiences, while the 14–15 age group exhibited no significant differences in comparison to adults. This analysis further supported differences in intensity responses to tenderness clips: participants aged 8–9, 10–11, and 12–13 rated clips in the tenderness category less intense as compared to adults (with a decrease in β values of 18.12, 14.60, and 10.69 respectively, all *p*s < 0.01). Furthermore, participants aged 8–9 (β = 10.69, *p* = .002), and 10–11 (β = 7.26, *p* = .044) reported more intense responses to the fear category compared to adults. There were no significant differences between the older groups.

Additional post-hoc analyses were conducted to determine differences among individual emotion categories of clips. Tenderness ratings were significantly less intense compared to joy (-22.45, *p* < .001), anger (-8.18, *p* < .001), sadness (-16.21, *p* < .001), and amusement (-13.77, *p* < .001). Conversely, clips evoking joy elicited higher intensity than those evoking anger (14.27, *p* < .001), sadness (6.25, *p* = .012), fear (20.05, *p* < .001), and amusement (8.68, *p* < .001). Intensity ratings for anger were significantly lower than those for sadness (-8.02, *p* < .001) and amusement (-5.59, *p* = .036) clips. Further details are available in the Supplementary Tables 7–8.

### Effects of age and emotion category on physiological responses

We next investigated whether the physiological responses to our video clips varied depending on age and emotion category (Fig. 2B-C, Supplementary Tables 9–10). For HR, the polynomial model was found to be the best fit, demonstrating a significant curvilinear relationship between age and HR (Age: F(1, 111) = 21.27, *p* < .001; Age^2^: F(1, 111) = 19.04, *p* < .001) and no significant effect of category.

The categorical model showed main effects of both age group (F(4, 109) = 4.09, *p* = .004) and emotion category (F(5, 545) = 26.64, *p* < .001). We identified a significantly greater HR response to fear (β = 0.791, *p* < .001) and a significantly smaller response to anger (β = -0.52, *p* = .004) across all age groups when compared to the mean of all other emotions. The interaction effect between age and emotion category was not significant (Supplementary Tables 11–13).

For HRV, the linear age model demonstrated a better fit than polynomial (Supplementary Tables 14–15), showing a significant decline in HRV with increasing age (F(1, 111.47) = 17.90, *p* < .001). However, the effects of emotion category and interaction between age and emotion category were not significant (Supplementary Table 16).

### Effects of age and emotion category on behavioural responses

Next, we examined the gaze behaviour (measured as the percentage of gaze on screen) across different age groups and emotion categories (Fig. 2D, Supplementary Tables 15–16). The linear age model revealed a significant main effect of emotion category (F(5, 563.97) = 27.55, *p* < .001) and an interaction between age and category (F(5, 563.97) = 5.08, *p* < .001). The main effect of age was not significant (F(1, 112.96) = 3.74, *p* = .056). The model showed that fear (β = -6.06, *p* < .001), tenderness (β = -2.32, *p* = .001), and sadness (β = -1.73, *p* = .015) were associated with a decrease in gaze on screen compared to mean of all emotions. In contrast, joy (β = 2.36, *p* < .001) and anger (β = 2.77, *p* < .001) were associated with increased gaze on screen, suggesting more sustained visual engagement. The analyses showed that the significant interaction effect was due to an increase in percentage of gaze on screen to fear with increasing age (β = 0.21, *p* < .001), suggesting a developmental change in visual engagement with fear over time.

The categorical age model showed main effects for both age group (F(4, 110.0) = 2.7, *p* = .034) and emotion category (F(5, 549.0) = 83.5, *p* < .001), and a significant interaction of age and emotion category (F(20, 549.0) = 1.8, *p* = .016). Post hoc comparisons of age groups revealed that, as compared to adults, the percentage of gaze on screen was significantly lower only in children in age group of 8–9 years (β = -5.43, *p* = .008) (Supplementary Tables 19–21).

### Multicomponent representational similarity analysis

**Fig. 3:**
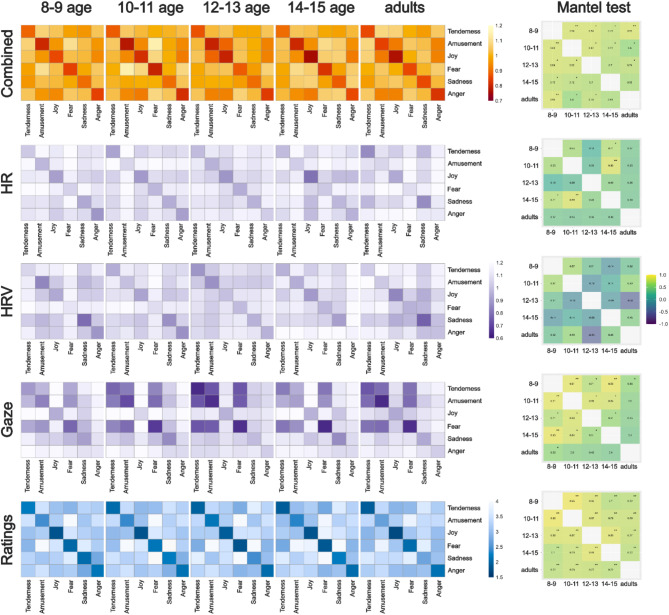
The multicomponent Representational Similarity Analysis (RSA). Similarity matrices for a combination of HR, HRV, and percentage of gaze on screen (top row), for each of the components separately, and for intensity ratings (bottom row) as a reference. The similarity matrices represent similarities in emotional responses between emotion categories (tenderness, amusement, joy, fear, sadness, anger) for each age group separately. Each cell in the similarity matrix represents the distance between the emotional responses to pairs of emotions. Lower values indicate greater similarity (i.e., more similar physiological responses), while higher values suggest greater dissimilarity (i.e., more distinct physiological responses). The matrix is symmetric, with the diagonal representing the similarity of reactions to clips within the same emotion category. The Mantel test figure represents the correlation between similarity matrices. The * indicates that there is a statistically significant correlation between the two matrices, *p < .05., **p < .01., ***p < .001.

We investigated the similarity of responses between different age groups for two components - the physiological (HR and HRV) and behavioural responses (percentage of gaze on screen) - together. Across all age groups, we observed that the similarity values within the same emotional category, as indicated by the diagonal of each RSA matrix, generally showed lower values (greater similarity) compared to those between different emotional categories (Fig. 3). Across all matrices, the mean diagonal values range from 0.83 to 0.85, while the mean off-diagonal values are consistently higher, ranging from 1.00 to 1.02. This suggests a distinct physiological and behavioural response pattern for each emotion was identifiable across all age groups.

Fear and anger seemed to elicit the most distinct responses across all age groups, indicated by the lowest similarity scores with other categories (within-group similarity scores ranged from 0.79 to 0.87 for fear and 0.80 to 0.86 for anger). Amusement (0.78–0.84) and joy (0.72–0.84) demonstrated moderate to high similarity scores across ages, suggesting a certain level of overlap in the physiological and behavioural responses to these emotions.

The analysis of inter-age group similarities using Mantel’s test demonstrated correlations between age groups, with values ranging from 0.60 to 0.85. The highest similarity scores were observed between young children and adults (0.85). However, this pattern is not uniform, as evidenced by the variability in similarity scores across different age comparisons.

The RSA results for each single component demonstrated that response patterns were identifiable only for a few emotion categories. Joy appeared to show a distinct HR response across all age groups, although the uniqueness of the anger response was evident only in the youngest group and diminished with age. In contrast to the children’s groups, adults appeared to exhibit the most distinct HR response pattern for clips inducing tenderness and sadness. The Mantel test revealed a significant correlation between the 8–9 age group and the 14–15 age group, as well as between the 10–11 age group and the 14–15 age group.

HRV response patterns were also identifiable for some categories. Sadness and joy categories showed the most distinct HRV response across all age groups. Similarly, the HRV response pattern to anger was pronounced in the youngest group and less so in the older groups. The adult group appeared to demonstrate similar HRV response patterns in response to negative emotion categories (anger, fear, sadness). The Mantel test did not show significant similarities between age groups.

The RSA analysis of gaze behaviour did not reveal distinguishable response patterns for emotion categories as the similarity values within the same emotional category showed similar values compared to those between different emotional categories.

**Fig. 4:**
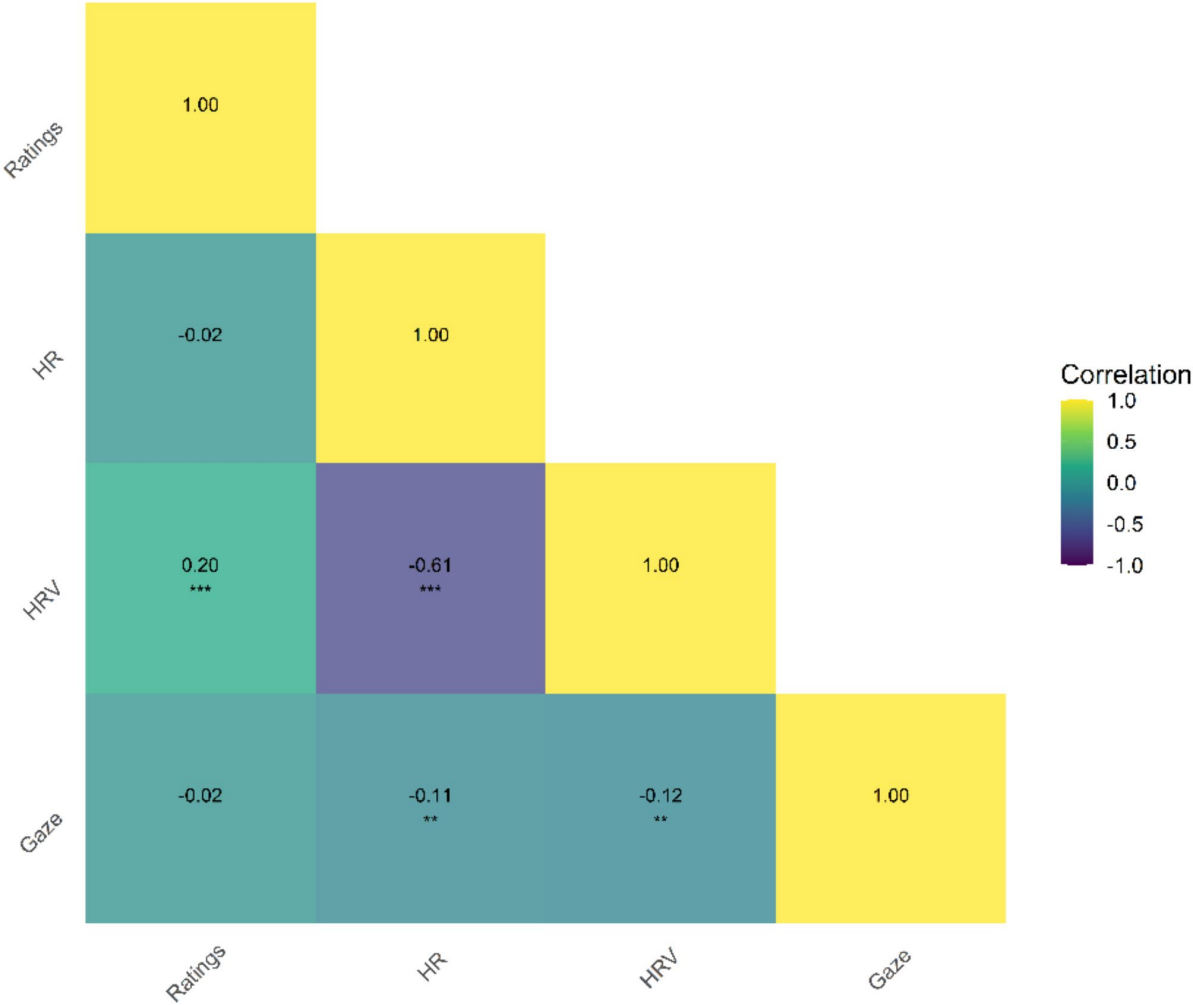
Correlation between components. Spearman correlations between measurements. *p < .05., **p < .01.,***p < .001.

Finally, we also compared similarity matrices of single components to each other (Fig. 4). As expected, physiological responses – HR and HRV had the highest correlation with each other. HRV also correlated with both ratings and percentage of gaze on screen. This provides evidence that single components convey partly unique information regarding emotion categories.

## Discussion

The study examined age-related responses to emotional video stimuli, focusing on three emotion components: experienced intensity, physiological responses, and gaze behaviour. Subsequently, we discuss the findings for each component.

### Subjective experiences

The results showed that emotional intensity ratings generally decreased with age, consistent with previous findings showing the tendency for young children to use extremes when rating emotional states^13,15,26^. Supplementary analysis showed that the average intensity of non-target emotions also decreased with age, mirroring the trend observed in the intensity of target emotions (Supplementary Tables 22–25). A plausible explanation is that younger children indeed experienced emotions more intensely than older individuals. Although children can accurately use scales to rate physical modalities, suggesting reliable scaling abilities, they often give extreme emotional ratings^15^. Older individuals might contextualise their emotions differently, utilising a broader range of past experiences in their ratings. This notion complements the idea that younger individuals may feel emotions more intensely due to fewer past experiences and less developed self-regulation^71^, making new encounters seem more impactful. The same stimulus material was used for all age groups, which may explain the lower intensity ratings in older participants. Furthermore, the changes of emotional ratings with age could involve an evolution from binary (e.g., angry or not) to more nuanced emotional evaluations. This is supported by research indicating a shift from unidimensional, valence-centric emotional representations to bidimensional frameworks considering both valence and arousal^72^, alongside the categorisation of basic emotions like fear, sadness, and happiness^73^.

Contrary to the general trend, tenderness ratings increased with age. Tenderness is not commonly recognised as a basic emotion^74^, and children might learn this term later than others^75^. Although participants were familiarised with the term, prior studies have shown that children’s comprehension of emotional vocabulary may not fully converge with adult interpretations^76^ and is often used in overgeneralised manner^77^.

### Physiological responses

Physiological responses showed an age-related curvilinear change for HR, consistent with typical cardiac maturation trends^41^. However, while HRV appears visually curvilinear in the figure, the polynomial age model did not provide a better fit than the linear model. This discrepancy may be due to the small number of adult participants over 21 years, whose higher HRV values contributed to increased variability at the upper end of the age distribution. To differentiate emotion-specific developmental changes from general physiological maturation, we included an age × emotion category interaction in our models. While emotional categories did not significantly affect HRV, fear-inducing stimuli prompted increased HR across all age groups, indicating a universal physiological arousal in response to fear^5,36,78,79^. Conversely, anger-inducing clips elicited lower HR across all participants. While previous research has shown mixed findings regarding the effect of anger on HR, Stemmler and colleagues^80^ suggested that motivation type influences HR response to anger, with withdrawal-linked anger decreasing HR.

HR and HRV responses were largely similar across emotion categories. While previous studies have found distinct behavioural, somatosensory, and neural responses for different emotion categories (for a review, see^4^), there is less evidence for a single autonomous nervous system activity that distinguishes between emotion categories (e.g^37,81^). Detecting distinct physiological patterns for different emotions may require analysing multiple physiological signals rather than relying solely on HR.

### Behavioural responses

Fear, sadness, and tenderness were related to less gaze on screen, particularly among younger participants. Changes in gaze on screen can result from either active aversion or less engagement. Less gaze on screen in relation to fear and sadness potentially stems from their distressing nature, prompting avoidance behaviours^82^. Although tenderness is considered a positive emotion, it is distinct from joy and is known to co-occur with sadness during pitiful scenes^74^. This duality was also reflected in behavioural ratings showing an overlap between tenderness and sadness. Conversely, stimuli eliciting anger increased visual engagement, suggesting that such emotional content captivated attention through engagement or confrontation. These observations aligned with previous findings, which posited that anger engaged an approach motivation system, while fear, anxiety, and sadness triggered avoidance^83,84^.

Children aged 8–11 displayed significantly less gaze on screen than adults, particularly in response to fear-inducing stimuli, potentially reflecting heightened sensitivity and robust avoidance behaviour. These age groups also reported higher emotional intensity ratings, reinforcing the notion of increased emotional reactivity. While the gaze aversion in younger children could be attributed to increased physical motion while viewing the clips, potentially signalling a lack of attentional control^71,85^ and evolving emotional coping strategies^86^, we found no correlation between age and motion - as denoted by increased noise in ECG signal - in our sample of 8-15-year-olds.

Finally, while the quality of eye-tracking data could potentially be compromised by long stimulus presentation, our participants did not report fatigue during the experiment. Naturalistic stimuli, such as movies, have been proposed to decrease motion in demanding experimental setting, such as during brain imaging^87^. Based on continuous monitoring by the research assistants, participants did not overtly express difficulties in engaging with the paradigm. This observation, combined with the lack of a relationship between age and motion, supports the use of movie stimuli when studying children.

### Multi-component approach

This study highlighted the importance of a multi-component approach in emotion research, demonstrating that different modalities collectively contributed essential insights that were inaccessible through isolated components. Notably, fear-inducing stimuli elicited comparatively low-intensity ratings, especially among older participants. However, we observed pronounced physiological and behavioural responses to such stimuli across all age groups. Furthermore, the data revealed no significant difference in the self-reported experience of fear between 14-15-year-olds and adults. However, adolescents’ physiological and behavioural responses were markedly more pronounced, evidenced by increased HR and greater gaze aversion when exposed to fearful stimuli. Similarly, while younger children (8–9 years) provided low tenderness ratings, their behavioural responses indicated heightened avoidance.

Relying on the component process model, we presented combined RSA metrics including physiological (HR, HRV) and behavioural (gaze) data. In contrast, self-reported intensity ratings, conceptualised here as the final component in emotional processing, were presented separately. The exploratory RSA analysis, incorporating HR, HRV, and gaze behaviour, revealed distinct multi-component response patterns unique to each emotion category, not evident when responses were analysed separately. In line with the component process model, the similarity of combined response patterns resembled that of the subjective ratings, supporting the subjective experience as a result of integration of other components^2^. Notably, across all age groups, fear-inducing stimuli elicited a response pattern that is clearly distinct from other emotion categories, highlighting its primal role in human emotional experience^78,79,88^. These findings advocate for the inclusion of multiple assessment methods to gain a comprehensive understanding of emotional experiences across different age groups.

#### Limitations

The current study is a cross-sectional study with a relatively low sample size, which might affect the generalisability of the findings, particularly in the RSA analysis and LMM group comparisons. The small sample size led us to group children into age categories, which is not optimal. Ideally, with a larger sample, we would have constructed RSA matrices for each year; however, due to our sample size, we grouped ages to ensure sufficient statistical power. While our findings reveal age-related trends in affective responses, the exploratory nature of the study means that these results serve as a foundation for generating hypotheses rather than providing definitive conclusions. Future studies with confirmatory designs are needed to validate and expand upon these observations.

Additionally, the homogeneity of the sample, particularly in terms of race, ethnicity, and socioeconomic background, limits the generalisability of our findings to more diverse populations. Another limitation pertains to the missing heart rate data, predominantly due to excessive movements in children, indicating the need to ensure participant comfort to reduce data loss due to motion. To assess the impact of missing data across all modalities, we conducted additional analyses examining whether data loss was systematically related to key variables such as age and emotion category. Chi-square tests showed that missing data were evenly distributed across emotion categories, while t-tests and further inspection of influential cases suggested that significant age-related differences were driven by individual cases rather than systematic bias. Given the small proportion of missing data (between 1 and 7 participants per modality), and its random distribution across key variables, we conclude that missing data is unlikely to have affected the overall pattern of results. Additional details can be found in Supplementary Materials (“Analysis of missing values”).

Furthermore, the gaze detection methodology did not allow for differentiation between blinks and instances of looking away in the gaze data, which limits the interpretation of the findings. It is possible that the age-related shift towards more gaze to fearful stimuli reflects either increasing salience of the stimuli or less data loss in older children and adolescents. Future studies should consider employing more sophisticated eye-tracking tools capable of distinguishing between these two types of visual behaviour.

In this study, participants of various ages were exposed to identical video stimuli, selected to be appropriate for the youngest participants (7 years old) to adhere to ethical standards. This age constraint likely influenced the appraisal of the videos, as developmental stages affect emotional processing and sensitivity to media content. Given these constraints, it is unlikely that participants across age groups experienced emotional responses of the same intensity or authenticity, as highlighted by the emotion ratings. We recognise that developmental relevance is crucial for understanding age-related emotional responses. A more robust approach could involve a longitudinal design, exposing the same participants to age-appropriate stimuli at different developmental stages. This would provide deeper insights into the evolution of emotional responses over time beyond what is possible with a cross-sectional design constrained by standardised stimuli.

## Conclusions

Our results demonstrated changes in self-reports, physiology, and behavioural responses to emotional stimuli from childhood to adulthood. Emotional intensity ratings and behavioural responses varied by emotion category and age, while physiological responses showed an age effect but did not distinguish between emotion categories. Furthermore, specific emotions showed different maturation patterns. From the methodological perspective, the study underscored the importance of integrating multiple physiological measures to discern emotional states accurately. The RSA analysis demonstrated that a singular measure may not sufficiently differentiate between emotion categories. In contrast, a combination of measures revealed more distinct emotional patterns, even in younger age groups.

## Electronic supplementary material

Below is the link to the electronic supplementary material.

Supplementary Material 1

## Data Availability

The anonymised data collected are available as open data via OFS online data repository: https://osf.io/82dz4/?view_only=a11e13fded5149569576588dd35fa4c4. The files also include R scripts used for the statistical analysis as presented in the manuscript. All video stimuli are available from the corresponding author on request.

## References

[1] Lane, R. D. R., Nadel, L. & Ahern, G. L. Cognitive neuroscience of emotion. series in affective science. (Oxford University Press, (2000).

[2] Sander, D., Grandjean, D. & Scherer, K. R. An Appraisal-Driven componential approach to the emotional brain. *Emot. Rev.* **10**, 219–231 (2018).

[3] Kragel, P. A. & LaBar, K. S. Decoding the nature of emotion in the brain. *Trends Cogn. Sci.* **20**, 444–455 (2016). 27133227 10.1016/j.tics.2016.03.011 PMC4875847

[4] Nummenmaa, L. & Saarimäki, H. Emotions as discrete patterns of systemic activity. *Neurosci. Lett.* **693**, 3–8 (2019). 28705730 10.1016/j.neulet.2017.07.012

[5] Anderson, D. J. & Adolphs, R. A framework for studying emotions across species. Cell vol. 157 187–200 at (2014). 10.1016/j.cell.2014.03.003 10.1016/j.cell.2014.03.003 PMC4098837 24679535

[6] Barrett, L. F., Mesquita, B., Ochsner, K. N. & Gross, J. J. The experience of emotion. *Annu. Rev. Psychol.* **58**, 373–403 (2007). 17002554 10.1146/annurev.psych.58.110405.085709 PMC1934613

[7] Cowen, A. S. & Keltner, D. Self-report captures 27 distinct categories of emotion bridged by continuous gradients. *Proc. Natl. Acad. Sci. U. S. A.* 114, E7900–E7909 (2017). 10.1073/pnas.1702247114 PMC5617253 28874542

[8] Goldin, P. R. et al. The neural bases of amusement and sadness: A comparison of block contrast and subject-specific emotion intensity regression approaches. *Neuroimage* **27**, 26–36 (2005). 15890534 10.1016/j.neuroimage.2005.03.018

[9] Horikawa, T., Cowen, A. S., Keltner, D. & Kamitani, Y. The neural representation of visually evoked emotion is high-dimensional, categorical, and distributed across transmodal brain regions. *iScience* 23, 101060 (2020). 10.1016/j.isci.2020.101060 PMC7191651 32353765

[10] Chan, H. Y., Smidts, A., Schoots, V. C., Sanfey, A. G. & Boksem, M. A. S. Decoding dynamic affective responses to naturalistic videos with shared neural patterns. *Neuroimage* **216**, 116618 (2020). 32036021 10.1016/j.neuroimage.2020.116618

[11] Nummenmaa, L. et al. Emotions promote social interaction by synchronizing brain activity across individuals. *Proc. Natl. Acad. Sci. U S A*. **109**, 9599–9604 (2012). 22623534 10.1073/pnas.1206095109 PMC3386135

[12] Durbin, C. E. Validity of young children’s self-reports of their emotion in response to structured laboratory tasks. *Emotion* **10**, 519–535 (2010). 20677869 10.1037/a0019008

[13] Gabel, L. N. et al. Development and validation of a battery of emotionally evocative film clips for use with young children. *Psychol. Assess.* **31**, 1040–1051 (2019). 31045383 10.1037/pas0000726

[14] Hietanen, J. K., Glerean, E., Hari, R. & Nummenmaa, L. Bodily maps of emotions across child development. *Dev. Sci.* **19**, 1111–1118 (2016). 26898716 10.1111/desc.12389

[15] Chambers, C. T. Developmental differences in children’s use of rating scales. *J. Pediatr. Psychol.* **27**, 27–36 (2002). 11726677 10.1093/jpepsy/27.1.27

[16] Zeman, J., Klimes-Dougan, B., Cassano, M. & Adrian, M. Measurement issues in emotion research with children and adolescents. Clinical Psychology: Science and Practice vol. 14 377–401 at (2007). 10.1111/j.1468-2850.2007.00098.x

[17] Sohn, J. H., Sokhadze, E. & Watanuki, S. Electrodermal and cardiovascular manifestations of emotions in children. *J. Physiol. Anthropol. Appl. Hum. Sci.* **20**, 55–64 (2001). 10.2114/jpa.20.55 11385939

[18] Wilhelm, F. H., Schneider, S. & Friedman, B. H. Psychophysiological assessment. in Clinician’s handbook of child behavioral assessment 201–231 (Elsevier Inc., doi:10.1016/B978-012343014-4/50010-1. (2006).

[19] Barrett, L. F., Adolphs, R., Marsella, S., Martinez, A. M. & Pollak, S. D. Emotional expressions reconsidered: challenges to inferring emotion from human facial movements. *Psychol. Sci. Public. Interes*. **20**, 1–68 (2019). 10.1177/1529100619832930 PMC6640856 31313636

[20] Witkower, Z. & Tracy, J. L. Bodily communication of emotion: evidence for extrafacial behavioral expressions and available coding systems. *Emot. Rev.* **11**, 184–193 (2019).

[21] Cowen, A. S., Elfenbein, H. A., Laukka, P. & Keltner, D. Mapping 24 emotions conveyed by brief human vocalization. *Am. Psychol.* **74**, 698–712 (2019). 30570267 10.1037/amp0000399 PMC6586540

[22] Skaramagkas, V. et al. Review of eye tracking metrics involved in emotional and cognitive processes. *IEEE Rev. Biomed. Eng.*10.1109/RBME.2021.3066072 (2021). 10.1109/RBME.2021.3066072 33729950

[23] Adams, R. B. & Kleck, R. E. Effects of direct and averted gaze on the perception of facially communicated emotion. *Emotion* **5**, 3–11 (2005). 15755215 10.1037/1528-3542.5.1.3

[24] Maffei, A. & Angrilli, A. Spontaneous Blink rate as an index of attention and emotion during film clips viewing. *Physiol. Behav.* **204**, 256–263 (2019). 30822434 10.1016/j.physbeh.2019.02.037

[25] Goshvarpour, A. & Goshvarpour, A. Eye-blinking analysis as a marker of emotional States. *Multimed Tools Appl.* **80**, 33727–33746 (2021).

[26] McManis, M. H., Bradley, M. M., Berg, W. K., Cuthbert, B. N. & Lang, P. J. Emotional reactions in children: verbal, physiological, and behavioral responses to affective pictures. *Psychophysiology* **38**, 222–231 (2001). 11347868

[27] Waters, A. M., Lipp, O. V. & Spence, S. H. The effects of affective picture stimuli on blink modulation in adults and children. *Biol. Psychol.* **68**, 257–281 (2005). 15620794 10.1016/j.biopsycho.2004.05.002

[28] Cuthbert, B. N., Bradleym, M. M. & Lang, P. J. Probing picture perception: activation and emotion. *Psychophysiology* **33**, 103–111 (1996). 8851238 10.1111/j.1469-8986.1996.tb02114.x

[29] Golland, Y. & Keissar, K. Levit-Binnun, N. Studying the dynamics of autonomic activity during emotional experience. *Psychophysiology* **51**, 1101–1111 (2014). 25039415 10.1111/psyp.12261

[30] Wallentin, M. et al. Amygdala and heart rate variability responses from listening to emotionally intense parts of a story. *Neuroimage* **58**, 963–973 (2011). 21749924 10.1016/j.neuroimage.2011.06.077

[31] Brouwer, A. M., Hogervorst, M., Reuderink, B., van der Werf, Y. & van Erp, J. Physiological signals distinguish between reading emotional and non-emotional sections in a novel. *Brain-Computer Interfaces*. **2**, 76–89 (2015).

[32] Nummenmaa, L. et al. Emotional speech synchronizes brains across listeners and engages large-scale dynamic brain networks. *Neuroimage* **102**, 498–509 (2014). 25128711 10.1016/j.neuroimage.2014.07.063 PMC4229500

[33] Eisenbarth, H., Chang, L. J. & Wager, T. D. Multivariate brain prediction of heart rate and skin conductance responses to social threat. *J. Neurosci.* **36**, 11987–11998 (2016). 27881783 10.1523/JNEUROSCI.3672-15.2016 PMC5125248

[34] Scheer, C., Kubowitsch, S., Dendorfer, S. & Jansen, P. Happy enough to relax?? How positive and negative emotions activate different muscular regions in the back - an explorative study. *Front. Psychol.* **12**, 2011 (2021). 10.3389/fpsyg.2021.511746 PMC8201496 34135791

[35] Joseph, N. T., Jiang, Y. & Zilioli, S. Momentary emotions and salivary cortisol: A systematic review and meta-analysis of ecological momentary assessment studies.* Neurosci. Biobehav.Rev* 125 365–379 at (2021). 10.1016/j.neubiorev.2021.02.042 10.1016/j.neubiorev.2021.02.042 33662445

[36] Kreibig, S. D. Autonomic nervous system activity in emotion: A review.* Biol. Psychol.* 84 394–421 at (2010). 10.1016/j.biopsycho.2010.03.010 10.1016/j.biopsycho.2010.03.010 20371374

[37] Siegel, E. H. et al. Emotion fingerprints or emotion populations? A meta-analytic investigation of autonomic features of emotion categories. *Psychol. Bull.* **144**, 343–393 (2018). 29389177 10.1037/bul0000128 PMC5876074

[38] Sacrey, L. R. et al. Physiological measurement of emotion from infancy to preschool: A systematic review and meta-analysis. *Brain Behav.* **11**, e01989 (2021). 33336555 10.1002/brb3.1989 PMC7882167

[39] Koch, A. & Pollatos, O. Cardiac sensitivity in children: sex differences and its relationship to parameters of emotional processing. *Psychophysiology* **51**, 932–941 (2014). 24810627 10.1111/psyp.12233

[40] Leventon, J. S., Stevens, J. S. & Bauer, P. J. Development in the neurophysiology of emotion processing and memory in school-age children. *Dev. Cogn. Neurosci.* **10**, 21–33 (2014). 25160677 10.1016/j.dcn.2014.07.007 PMC6987950

[41] Harteveld, L. M. et al. Maturation of the cardiac autonomic nervous system activity in children and adolescents. *J. Am. Heart Assoc.* **10**, 1–22 (2021). 10.1161/JAHA.120.017405 PMC7955328 33525889

[42] Mauss, I. B., McCarter, L., Levenson, R. W., Wilhelm, F. H. & Gross, J. J. The tie that binds? Coherence among emotion experience, behavior, and physiology. *Emotion* **5**, 175–190 (2005). 15982083 10.1037/1528-3542.5.2.175

[43] Scherer, K. R. & Moors, A. The emotion process: event appraisal and component differentiation. *Annu. Rev. Psychol.* **70**, 719–745 (2019). 30110576 10.1146/annurev-psych-122216-011854

[44] Fiskum, C. et al. Reactive heart rate variability and cardiac entropy in children with internalizing disorder and healthy controls. *Appl. Psychophysiol. Biofeedback*. **44**, 309–319 (2019). 31300950 10.1007/s10484-019-09444-0

[45] Gilissen, R., Bakermans-Kranenburg, M. J., van IJzendoorn, M. H. & van der Veer, R. Parent-child relationship, temperament, and physiological reactions to fear-inducing film clips: further evidence for differential susceptibility. *J. Exp. Child. Psychol.* **99**, 182–195 (2008). 17681350 10.1016/j.jecp.2007.06.004

[46] von Leupoldt, A. et al. Films for eliciting emotional states in children. *Behav. Res. Methods*. **39**, 606–609 (2007). 17958174 10.3758/bf03193032

[47] Davis, E. L., Quiñones-Camacho, L. E. & Buss, K. A. The effects of distraction and reappraisal on children’s parasympathetic regulation of sadness and fear. *J. Exp. Child. Psychol.* **142**, 344–358 (2016). 26601786 10.1016/j.jecp.2015.09.020 PMC4666770

[48] De Wied, M., Boxtel, A., Van, Posthumus, J. A., Goudena, P. P. & Matthys, W. Facial EMG and heart rate responses to emotion-inducing film clips in boys with disruptive behavior disorders. *Psychophysiology* **46**, 996–1004 (2009). 19549069 10.1111/j.1469-8986.2009.00851.x

[49] Lebert, A., Chaby, L., Garnot, C. & Vergilino-Perez, D. The impact of emotional videos and emotional static faces on postural control through a personality trait approach. *Exp. Brain Res.* **238**, 2877–2886 (2020). 33057868 10.1007/s00221-020-05941-5

[50] Sun, Y., Ayaz, H. & Akansu, A. N. Multimodal affective state assessment using fNIRS + EEG and spontaneous facial expression. *Brain Sci.* **10**, 85 (2020). 32041316 10.3390/brainsci10020085 PMC7071625

[51] Saarimäki, H. Naturalistic stimuli in affective neuroimaging: A review. *Front. Hum. Neurosci.* **15**, (2021). 10.3389/fnhum.2021.675068 PMC8245682 34220474

[52] Scherer, K. R. The dynamic architecture of emotion: evidence for the component process model. *Cogn. Emot.* **23**, 1307–1351 (2009).

[53] Barrett, L. F. Are emotions natural kinds?? *Perspect. Psychol. Sci.* **1**, 28–58 (2006). 26151184 10.1111/j.1745-6916.2006.00003.x

[54] Russell, J. A. A circumplex model of affect. *J. Pers. Soc. Psychol.* **39**, 1161–1178 (1980).

[55] Ekman, P. An argument for basic emotions. *Cogn. Emot.* **6**, 169–200 (1992).

[56] Tracy, J. L. & Randles, D. Four models of basic emotions: A review of Ekman and Cordaro, Izard, Levenson, and Panksepp and Watt. *Emot. Rev.* **3**, 397–405 (2011).

[57] Hamann, S. Mapping discrete and dimensional emotions onto the brain: controversies and consensus. *Trends Cogn. Sci.* **16**, 458–466 (2012). 22890089 10.1016/j.tics.2012.07.006

[58] Lindquist, K. A., Siegel, E. H., Quigley, K. S. & Barrett, L. F. The hundred-year emotion war: are emotions natural kinds or psychological constructions? Comment on Lench, Flores, and bench (2011). *Psychol. Bull.* **139**, 255–263 (2013). 23294094 10.1037/a0029038 PMC3556454

[59] Wood, A. & Coan, J. A. Beyond nature versus nurture: the emergence of emotion. *Affect. Sci.* **4**, 443–452 (2023). 37744982 10.1007/s42761-023-00212-2 PMC10513962

[60] Ekman, P. & Friesen, W. V. Constants across cultures in the face and emotion. *J. Pers. Soc. Psychol.* **17**, 124–129 (1971). 5542557 10.1037/h0030377

[61] Sauter, D. More than happy: the need for disentangling positive emotions. *Curr. Dir. Psychol. Sci.* **19**, 36–40 (2010).

[62] Keltner, D. & Cowen, A. A taxonomy of positive emotions. *Curr. Opin. Behav. Sci.* **39**, 216–221 (2021).

[63] Weidman, A. C. & Tracy, J. L. A provisional taxonomy of subjectively experienced positive emotions. *Affect. Sci.* **1**, 57–86 (2020). 36042965 10.1007/s42761-020-00009-7 PMC9382948

[64] Cowen, A., Sauter, D., Tracy, J. L. & Keltner, D. Mapping the passions: toward a High-Dimensional taxonomy of emotional experience and expression. *Psychol. Sci. Public. Interes*. **20**, 69–90 (2019). 10.1177/1529100619850176 PMC6675572 31313637

[65] Saarimäki, H. et al. Distributed affective space represents multiple emotion categories across the human brain. *Soc. Cogn. Affect. Neurosci.* **13**, 471–482 (2018). 29618125 10.1093/scan/nsy018 PMC6007366

[66] Saarimäki, H. et al. Cerebral topographies of perceived and felt emotions.* Imaging Neurosci*. (in press). 10.1162/imag_a_00517 PMC12319791 40800968

[67] Anwyl-Irvine, A. L., Massonnié, J., Flitton, A., Kirkham, N. & Evershed, J. K. Gorilla in our midst: an online behavioral experiment builder. *Behav. Res. Methods*. **52**, 388–407 (2020). 31016684 10.3758/s13428-019-01237-x PMC7005094

[68] Makowski, D. et al. NeuroKit2: A python toolbox for neurophysiological signal processing. *Behav. Res. Methods*. **53**, 1689–1696 (2021). 33528817 10.3758/s13428-020-01516-y

[69] Shaffer, F. & Ginsberg, J. P. An overview of heart rate variability metrics and norms. *Front. Public. Heal*. **5**, 258 (2017). 10.3389/fpubh.2017.00258 PMC5624990 29034226

[70] Kriegeskorte, N., Mur, M. & Bandettini, P. Representational similarity analysis - connecting the branches of systems neuroscience. *Front. Syst. Neurosci.* **2**, 249 (2008). 10.3389/neuro.06.004.2008 PMC2605405 19104670

[71] Posner, M. I. & Rothbart, M. K. Developing mechanisms of self-regulation. *Dev. Psychopathol.* **12**, 427–441 (2000). 11014746 10.1017/s0954579400003096

[72] Nook, E. C., Sasse, S. F., Lambert, H. K., McLaughlin, K. A. & Somerville, L. H. Increasing verbal knowledge mediates development of multidimensional emotion representations. *Nat. Hum. Behav.* **2017 112** (1), 881–889 (2017). 10.1038/s41562-017-0238-7 PMC5790154 29399639

[73] Grosse, G., Streubel, B., Gunzenhauser, C. & Saalbach, H. Let’s talk about emotions: the development of children’s emotion vocabulary from 4 to 11 years of age. *Affect. Sci.* **2**, 150–162 (2021). 36043167 10.1007/s42761-021-00040-2 PMC9382957

[74] Kalawski, J. P. Is tenderness a basic emotion? *Motiv Emot.* **34**, 158–167 (2010).

[75] Baron-Cohen, S., Golan, O., Wheelwright, S., Granader, Y. & Hill, J. Emotion word comprehension from 4 to 16 years old: A developmental survey. *Front. Evol. Neurosci.* **2**, 109 (2010). 21151378 10.3389/fnevo.2010.00109 PMC2996255

[76] Nook, E. C. et al. Charting the development of emotion comprehension and abstraction from childhood to adulthood using observer-rated and linguistic measures. *Emotion* **20**, 773–792 (2020). 31192665 10.1037/emo0000609 PMC6908774

[77] Widen, S. C. & Russell, J. A. Children acquire emotion categories gradually. *Cogn. Dev.* **23**, 291–312 (2008).

[78] LeDoux, J. E. & Hofmann, S. G. The subjective experience of emotion: a fearful view. Current opinion in behavioral dciences vol. 19 67–72 at (2018). 10.1016/j.cobeha.2017.09.011 10.1016/j.cobeha.2017.09.011 PMC11444666 39355512

[79] Panksepp, J. & Watt, D. What is basic about basic emotions?? Lasting lessons from affective neuroscience. (2011). 10.1177/1754073911410741 3, 387–396

[80] Stemmler, G., Aue, T. & Wacker, J. Anger and fear: separable effects of emotion and motivational direction on somatovisceral responses. *Int. J. Psychophysiol.* **66**, 141–153 (2007). 17544534 10.1016/j.ijpsycho.2007.03.019

[81] Kragel, P. A. & LaBar, K. S. Multivariate pattern classification reveals autonomic and experiential representations of discrete emotions. *Emotion* **13**, 681–690 (2013). 23527508 10.1037/a0031820 PMC3745776

[82] Elliot, A. J., Eder, A. B. & Harmon-Jones, E. Approach–avoidance motivation and emotion: convergence and divergence. (2013). 10.1177/1754073913477517 5, 308–311

[83] Carver, C. S. & Harmon-Jones, E. Anger is an rpproach-related affect: evidence and implications. *Psychol. Bull.* **135**, 183–204 (2009). 19254075 10.1037/a0013965

[84] Harmon-Jones, E. Anger and the behavioral approach system. *Pers. Individ Dif*. **35**, 995–1005 (2003).

[85] Kochanska, G., Murray, K. T. & Harlan, E. T. Effortful control in early childhood: continuity and change, antecedents, and implications for social development. *Dev. Psychol.* **36**, 220–232 (2000). 10749079

[86] Compas, B. E. et al. Coping, emotion regulation, and psychopathology in childhood and adolescence: A meta-analysis and narrative review. *Psychol. Bull.* **143**, 939–991 (2017). 28616996 10.1037/bul0000110 PMC7310319

[87] Vanderwal, T., Eilbott, J. & Castellanos, F. X. Movies in the magnet: naturalistic paradigms in developmental functional neuroimaging. *Dev. Cogn. Neurosci.* **36**, 100600 (2019). 30551970 10.1016/j.dcn.2018.10.004 PMC6969259

[88] Mobbs, D. et al. Viewpoints: approaches to defining and investigating fear. *Nat. Neurosci.* **22**, 1205–1216 (2019). 31332374 10.1038/s41593-019-0456-6 PMC6943931

