# Supplementary material for: Multicomponent approach reveals differences in affective responses among children and adolescents
Source: Sci Rep. 2025 Mar 25;15:10179. doi: 10.1038/s41598-025-94309-2 (PMC11933308; doi:10.1038/s41598-025-94309-2)
Supplement: Supplementary file 1 — Supplementary Material 1 [file 41598_2025_94309_MOESM1_ESM.docx]

**Multicomponent approach reveals differences in affective responses among children and adolescents**

*Kseniia Konopkina^1,2^, Hilla Hirvaskoski^1^, Jari K. Hietanen^1^, Heini Saarimäki^1^*

1. Human Information Processing Laboratory, Faculty of Social Sciences, Tampere University, Finland
2. Department of Psychology, University of Otago, Dunedin 9016, New Zealand

Corresponding author: Heini Saarimäki ([heini.saarimaki@tuni.fi](mailto:heini.saarimaki@tuni.fi))

**Contents**

[Supplementary figures 1](#_heading=h.30j0zll)

[Supplementary tables 2](#_heading=h.3znysh7)

[Supplementary analyses 20](#_heading=h.2et92p0)

[*Linear mixed-effects models for non-target emotion 20*](#_heading=h.tyjcwt)

[*Linear mixed-effects models for experienced emotions 23*](#_heading=h.3dy6vkm)

## Supplementary figures

**Supplementary Figure 1**

*The rating interface used to capture participants' emotional experiences*


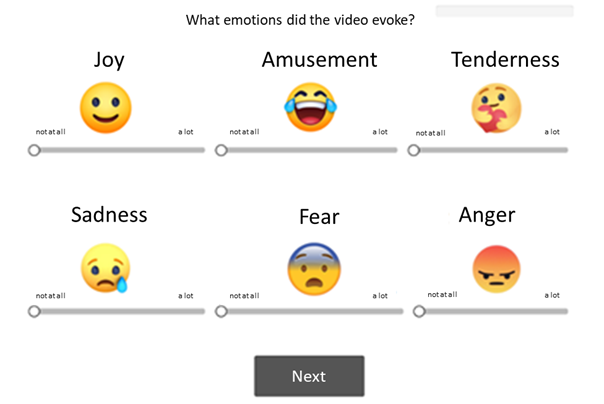


*Note.* The emotional rating interface was implemented using the Gorilla Experiment Builder (www.gorilla.sc; Anwyl-Irvine et al., 2020). In the experiment, all text labels were in Finnish.

## Supplementary tables

**Supplementary table 1**

*Results of the pre-evaluation survey for selecting clips that represent the target emotion category*

| **Video** | **Video name** | **Ratings** | **CATEGORY** | **Emotions (Finnish)** | **Emotions (English)** | **Int Mean** | **Int SD** | **Int Min** | **Int Max** |
| --- | --- | --- | --- | --- | --- | --- | --- | --- | --- |
| 120 | Kanelia kainaloon, Tatu ja Patu! | 1 | AMUSEMENT | huvittuneisuus | amusement | 7,00 | 0,00 | 7 | 7 |
| 127 | Heinähattu ja Vilttitossu | 3 | AMUSEMENT | suuttumus, huvittuneisuus (2), ärsyyntyminen | anger, amusement (2), irritation | 6,33 | 1,25 | 5 | 8 |
| 76 | First Day - 2020-09-07 - Jakso 02 - Yökyläilyä | 3 | AMUSEMENT | huvittuneisuus (3), ahdistus, ilo, hauskuus | amusement (3), anxiety, joy, funny | 5,00 | 2,16 | 3 | 8 |
| 80 | Kanelia kainaloon, Tatu ja Patu! | 3 | AMUSEMENT | huvittuneisuus (2), hämmennys, ilo, kauhu, järkytys, helpotus | amusement (2), confusion, joy, horror, shock, relief | 3,67 | 1,70 | 2 | 6 |
| 82 | Kanelia kainaloon, Tatu ja Patu! | 4 | AMUSEMENT | huvittuneisuus (3), hämmennys, hyväntuulisuus | amusement (3), confusion, joy | 3,00 | 1,73 | 2 | 6 |
| 1 | Mitä jos- - 2020-11-24 - Mitä jos.. haluaisi vielä leikkiä- (001) | 4 | AMUSEMENT | huvittuneisuus (2), turhautuneisuus, empatia, ilo | amusement (2), frustration, empathy, joy | 2,75 | 1,09 | 1 | 4 |
| 131 | Reetta & Ronja | 1 | AMUSEMENT | innostus, huvittuneisuus | excitement, amusement | 6 | 0 | 6 | 6 |
| 17 | Katto (short film) | 4 | AMUSEMENT | NONE, kiusaantuneisuus, uteliaisuus/mielenkiinto (2), huvittuneisuus | NONE, awkwardness, interest (2), amusement | 3,50 | 2,60 | 0 | 6 |
| 113 | Onneli, Anneli ja salaperäinen muukalainen - jakso 1 - Ruusukujan uudet asukkaat | 1 | ANGER | vihaisuus, ärsytys | anger, irritation | 7,00 | 0,00 | 7 | 7 |
| 13 | Ollaan kavereita - 2020-11-18 - Alfred ja kepposkerho | 4 | ANGER | viha/suuttumus (3), myötätunto, ärsytys, epäreiluus, huoli | anger (3), empathy, irritation, unfairness, worry | 6,75 | 2,17 | 3 | 8 |
| 109 | Jälki, joka jää - Eetu ja jäljet | 1 | ANGER | harmitus, vihaisuus | upset, anger | 6,00 | 0,00 | 6 | 6 |
| 88 | Keppi | 2 | ANGER | ärtymys (2), turhautuminen (2), viha | irritation (2), frustration (2), anger | 5,67 | 2,05 | 3 | 8 |
| 128 | Heinähattu ja Vilttitossu | 1 | ANGER | kaiho, vihaisuus, harmitus | TENDERNESS, anger, upset | 5,50 | 2,50 | 3 | 8 |
| 19 | Heinähattu ja Vilttitossu | 3 | ANGER / FEAR | suuttumus/viha (2), pelko (2), hämmästys, turhautuneisuus, empatia, suru, harmitus | anger (2), fear (2), surprise, frustration, empathy, sadness, upset | 6,67 | 1,25 | 5 | 8 |
| 103 | Avoin tarina - Uusperhe | 3 | ANGER / SADNESS / FEAR | pelko (2), inho, jännitys, suru (2), harmitus, vihaisuus, turhautuminen | fear (2), disgust, thrill, sadness (2), upset, anger, frustration | 5,67 | 0,47 | 5 | 6 |
| 92 | Avoin tarina - Lumisodan kuningas | 2 | ANGER | harmi, avuttomuus, epäoikeudenmukaisuus, suru, vihaisuus, pettymys, pelko, harmitus | irritation, helplessness, unfairness, sadness, anger, disappointment, fear, upset | 6,00 | 2,00 | 4 | 8 |
| 83 | Kanelia kainaloon, Tatu ja Patu! | 4 | ANGER | pettymys, hämmästys (2), ärsyyntyminen, turhautuminen, harmitus, huvittuneisuus | disappointment, surprise (2), irritation, frustration, upset, amusement | 4,50 | 2,29 | 1 | 7 |
| 98 | Avoin tarina - Jimi | 2 | ANGER | myötätunto, vihaisuus, harmitus, turhautuminen | empathy, anger, upset, frustration | 4,00 | 2,00 | 2 | 6 |
| 8 | Itch - 2020-09-01 - Kausi 01 - Jakso 01 - Kummallinen kivi | 2 | ANGER | jännitys, ärsytys, järkytys, vihaisuus | thrill, irritation, shock, anger | 3,50 | 2,50 | 1 | 6 |
| 114 | Onneli, Anneli ja salaperäinen muukalainen - jakso 1 - Ruusukujan uudet asukkaat | 1 | FEAR | jännitys, pelko, helpotus | anxiety, fear, relief | 6,00 | 0,00 | 6 | 6 |
| 12 | Itch - 2020-09-01 - Kausi 01 - Jakso 02 - Uusi tulokas | 2 | FEAR | jännitys (2), pelko | thrill (2), fear | 5,00 | 2,00 | 3 | 7 |
| 84 | Me Rosvolat | 3 | FEAR | jännitys (3), pelko (2) | thrill (3), fear (2) | 5,00 | 2,16 | 2 | 7 |
| 115 | Onneli, Anneli ja salaperäinen muukalainen - jakso 1 - Ruusukujan uudet asukkaat | 1 | FEAR | jännitys, pelko | anxiety, fear | 4,00 | 0,00 | 4 | 4 |
| 117 | Ihan sama - jakso 2 - Alin taju | 3 | FEAR | jännitys (3), hämmästys | thrill (3), surprise | 4,00 | 1,63 | 2 | 6 |
| 112 | Soppalinnan salaseura: Kellotorni | 2 | FEAR | innostus, jännitys (2), pelko | excitement, anxiety (2), fear | 3,50 | 0,50 | 3 | 4 |
| 116 | Ihan sama - jakso 1 - Kuka sinä olet | 2 | FEAR | jännitys (2), vaivaantuneisuus, pelko | anxiety (2), awkwardness, fear | 3,50 | 0,50 | 3 | 4 |
| 111 | Soppalinnan salaseura: Pako | 3 | FEAR | jännitys (3), hämmästys, hirvitys | thrill (3), confusion, fear | 3,33 | 2,62 | 1 | 7 |
| 118 | Ihan sama - jakso 2 - Alin taju | 2 | FEAR | jännitys (2), pelko | anxiety (2), fear | 2,50 | 1,50 | 1 | 4 |
| 2 | Mitä jos- - 2020-11-24 - Mitä jos.. nukahtaa julkiseen kulkuneuvoon- | 3 | FEAR | hämmästys, hämmennys, pelko, kauhu, epätoivo | surprise, confusion, fear, horror, despair | 2,00 | 0,82 | 1 | 3 |
| 93 | Avoin tarina - Joskus haluaisi vain huutaa | 3 | FEAR? | hämmästys (2), pelko, huoli, toivo | surprise (2), fear, worry, hope | 4,67 | 1,25 | 3 | 6 |
| 125 | Heinähattu ja Vilttitossu | 3 | JOY | ilo (3), huvittuneisuus, innostus (2) | joy (3), amusement, excitement (2) | 6,67 | 0,47 | 6 | 7 |
| 106 | Jälki, joka jää - Thimblin ja valta | 1 | JOY | ilo, onni | joy, happiness | 6,00 | 0,00 | 6 | 6 |
| 72 | First Day - 2020-09-07 - Jakso 01 - Eka päivä | 3 | JOY | ilo (2), vaivaantuneisuus, kyynisyys, onnellisuus, yllättyneisuus | joy (2), awkwardness, cynicism, happiness, surprise | 5,00 | 1,41 | 3 | 6 |
| 126 | Heinähattu ja Vilttitossu | 2 | JOY | ilo (2), innostus | joy (2), excitement | 5,00 | 2,00 | 3 | 7 |
| 85 | Me Rosvolat | 3 | JOY | ilo (2), rauhallisuus, onnellisuus, harmitus, helpotus | joy (2), calmness, happiness, upset, relief | 4,67 | 1,70 | 3 | 7 |
| 90 | Avoin tarina - Yksinäinen Valdemar | 2 | JOY | ilo (2), jännitys, ylpeys | joy (2), anxiety, pride | 4,50 | 1,50 | 3 | 6 |
| 91 | Avoin tarina - Lumisodan kuningas | 3 | JOY | ilo (2), ärsytys, hauskuus | joy (2), irritation, funny | 4,00 | 1,63 | 2 | 6 |
| 86 | Me Rosvolat | 4 | JOY | ilo (2), hämmästys, kaiho, jännitys, myötätunto, suru | joy (3), surprise, tenderness, thrill, empathy, sadness | 3,50 | 1,66 | 2 | 6 |
| 121 | Onnelin ja Annelin talvi | 1 | JOY | ilo, jännitys, hämmästys | joy, anxiety, surprise | 6,00 | 0,00 | 6 | 6 |
| 129 | Enni Intiassa | 1 | JOY | ilo, läheisyys, ihmetys | joy, togetherness, surprise | 4 | 0 | 4 | 4 |
| 130 | Hyvän kierrättäjät: Fillari | 1 | JOY | ilo, huvittuneisuus | joy, amusement | 4 | 0 | 4 | 4 |
| 122 | Onnelin, Anneli ja salaperäinen muukalainen - jakso 4 - Huolestuneet naapurit | 2 | JOY | ilo, ristiriita, hämmennys | joy, conflict, confusion | 3,50 | 2,50 | 1 | 6 |
| 101 | Avoin tarina - Jimi | 3 | TENDERNESS | myötätunto, haikeus, helpotus, toivo (2) | empathy, tenderness, relief, hope (2) | 6,00 | 1,63 | 4 | 8 |
| 123 | Onnelin, Anneli ja salaperäinen muukalainen - jakso 5 - Uusi suunnitelma | 3 | TENDERNESS | haikeus (2), ilo (2), hämmennys | tenderness (2), joy (2), confusion | 5,33 | 0,94 | 4 | 6 |
| 81 | Kanelia kainaloon, Tatu ja Patu! | 3 | TENDERNESS | haikeus, odotus, pettymys, pelko, jännitys, helpotus, hämmästys | tenderness, expectation, disappointment, fear, anxiety, relief, surprise | 3,67 | 1,70 | 2 | 6 |
| 119 | Ihan sama - jakso 2 - Alin taju | 3 | TENDERNESS | ilo, suru, haikeus, ihmetys | joy, sadness, tenderness, surprise | 2,67 | 1,25 | 1 | 4 |
| 124 | Heinähattu ja Vilttitossu | 2 | TENDERNESS | ilo, kaiho, harmitus, suru | joy, tenderness, upset, sadness | 5,50 | 1,50 | 4 | 7 |
| 102 | Avoin tarina - Uusperhe | 2 | TENDERNESS | hyväntuulisuus, suru, haikeus | joy, sadness, tenderness | 4,00 | 2,00 | 2 | 6 |
| 108 | Jälki, joka jää - Eetu ja jäljet | 1 | SADNESS | harmitus, suru, pettymys | upset, sadness, disappointment | 8,00 | 0,00 | 8 | 8 |
| 89 | Avoin tarina - Yksinäinen Valdemar | 3 | SADNESS | suru (3), viha/suuttumus (3), myötätunto, harmitus | sadness (3), anger (3), empathy, upset | 7,33 | 0,47 | 7 | 8 |
| 105 | Jälki, joka jää - Eemil ja erilaisuus | 1 | SADNESS | harmitus, suru | upset, sadness | 6,50 | 1,50 | 5 | 8 |
| 107 | Jälki, joka jää - Thimblin ja valta | 1 | SADNESS | harmitus, suru | upset, sadness | 6,00 | 0,00 | 6 | 6 |
| 97 | Avoin tarina - Joskus haluaisi vain huutaa | 3 | SADNESS | suru (3), epätoivo | sadness (3), despair | 5,33 | 2,05 | 3 | 8 |
| 100 | Avoin tarina - Jimi | 2 | SADNESS | avuttomuus, suru (2), haikeus, harmitus | helplessness, sadness (2), tenderness, upset | 5,00 | 2,00 | 3 | 7 |
| 87 | Me Rosvolat | 4 | SADNESS | haikeus (3), suru (2) | tenderness (3), sadness (2) | 4,75 | 1,30 | 3 | 6 |
| 95 | Avoin tarina - Joskus haluaisi vain huutaa | 3 | SADNESS | suru (3), haikeus (2) | sadness (3), tenderness (2) | 4,33 | 1,25 | 3 | 6 |
| 18 | Ajatuksia rakkaudesta (short film) | 4 | SADNESS | suru (3), toivo, hämmästys, harmitus | sadness (3), hope, surprise, upset | 4,00 | 1,87 | 2 | 7 |
| 99 | Avoin tarina - Jimi | 3 | SADNESS | suru (3), jännitys, myötätunto, harmitus, toivo | sadness (3), thrill, empathy, upset, hope | 4,00 | 0,00 | 4 | 4 |
| 104 | Jälki, joka jää - Iina ja ulkopuolisuus | 2 | SADNESS | ahdistus, suru (2), toivottomuus, kaiho, harmitus, vihaisuus | anxiety, sadness (2), despair, tenderness, upset, anger | 4,00 | 0,00 | 4 | 4 |
| 96 | Avoin tarina - Joskus haluaisi vain huutaa | 2 | SADNESS | myötätunto, suru, epätoivo, harmitus | empathy, sadness, despair, upset | 4,50 | 2,50 | 2 | 7 |
| 94 | Avoin tarina - Joskus haluaisi vain huutaa | 2 | SADNESS | suru, huolestuneisuus, pelko, jännitys, turhautuminen | sadness, worry, fear, anxiety, frustration | 3,50 | 2,50 | 1 | 6 |
| 110 | Soppalinnan salaseura: Pako | 2 |  | hämmennys, rohkeus, jännitys | confusion, courage, anxiety | 3,50 | 2,50 | 1 | 6 |

**Supplementary table 2**

*Video clips used in the video task. Familiarity is the percentage of participants who reported seeing the original movie or TV series before.*

| **Video set** | **ID** | **Duration** | **Familiarity** | **Content** | **Video name** |
| --- | --- | --- | --- | --- | --- |
| 1 | AMUSEMENT_01 | 00:25 | 31 % | Friends laughing and teasing each other | Mitä jos.. haluaisi vielä leikkiä |
| 2 | AMUSEMENT_02 | 01:06 | 83 % | Gingerbread crisis | Kanelia kainaloon, Tatu ja Patu! |
| 3 | AMUSEMENT_03 | 00:39 | 83 % | Tram | Kanelia kainaloon, Tatu ja Patu! |
| 4 | AMUSEMENT_04 | 01:03 | 83 % | Phone | Kanelia kainaloon, Tatu ja Patu! |
| 1 | AMUSEMENT_05 | 00:33 | 75 % | Weight lifting | Risto Räppääjä ja pullistelija |
| 5 | AMUSEMENT_06 | 00:42 | 75 % | Getting ready for sports | Risto Räppääjä ja pullistelija |
| 6 | AMUSEMENT_07 | 00:33 | 65 % | Man escapes | Risto Räppääjä ja väärä Vincent |
| 1 | ANGER_02 | 01:06 | 5 % | Argument between a child and a parent | Keppi |
| 2 | ANGER_03 | 00:44 | 14 % | Discussing bullying | Jälki, joka jää - Eetu ja jäljet |
| 2 | ANGER_04 | 00:23 | 78 % | Argument between a child and an adult | Onneli, Anneli ja salaperäinen muukalainen - Jakso 1 - Ruusukujan uudet asukkaat |
| 4 | ANGER_05 | 00:56 | 85 % | Sibling rivalry | Heinähattu ja Vilttitossu |
| 5 | ANGER_06 | 01:05 | 0 % | Thief steals a bag | Itch - Jakso 01 - Kummallinen kivi |
| 6 | ANGER_07 | 00:44 | 0 % | Tension between a pupil and a teacher | Avoin tarina - Jimi |
| 3 | ANGER_08 | 00:56 | 85 % | Sibling violence | Heinähattu ja Vilttitossu |
| 3 | FEAR_01 | 00:18 | 0 % | Stranger enters home | Itch - Kausi 01 - Jakso 02 - Uusi tulokas |
| 1 | FEAR_02 | 00:42 | 71 % | Escaping in a dark forest | Me Rosvolat |
| 3 | FEAR_03 | 00:21 | 6 % | Climbing high | Soppalinnan salaseura: Kellotorni |
| 2 | FEAR_04 | 00:47 | 78 % | Walking in a dark forest | Onneli, Anneli ja salaperäinen muukalainen - Jakso 1 - Ruusukujan uudet asukkaat |
| 4 | FEAR_05 | 00:57 | 17 % | fear of consequences | Ihan sama - Jakso 1 - Kuka sinä olet |
| 5 | FEAR_06 | 00:55 | 17 % | Escaping | Ihan sama - Jakso 2 - Alin taju |
| 6 | FEAR_08 | 00:58 | 78 % | fear of consequences | Onneli, Anneli ja salaperäinen muukalainen - Jakso 1 - Ruusukujan uudet asukkaat |
| 1 | JOY_01 | 00:31 | 8 % | Family exchanging gifts | First Day - Jakso 01 - Eka päivä |
| 3 | JOY_02 | 00:48 | 8 % | Soccer skills on school yard | Jamie Johnson - Jakso 01 - Koulun vaihtaminen |
| 2 | JOY_03 | 01:06 | 85 % | Family hugging and playing | Heinähattu ja Vilttitossu |
| 5 | JOY_04 | 01:00 | 85 % | Family playing | Heinähattu ja Vilttitossu |
| 4 | JOY_05 | 00:46 | 8 % | Dancing contest | First Day - Jakso 02 - Yökyläilyä |
| 6 | JOY_06 | 00:46 | 12 % | Fixing a bike together | Hyvän kierrättäjät |
| 4 | JOY_07 | 00:48 | 5 % | Father and son sled riding | Esteitä ja ylityksiä: Ikävä |
| 1 | SADNESS_01 | 00:25 | 5 % | Boy crying for a broken heart | Ajatuksia rakkaudesta |
| 3 | SADNESS_02 | 00:52 | 71 % | Saying goodbye | Me Rosvolat |
| 4 | SADNESS_03 | 01:10 | 0 % | Son leaving mother | Avoin tarina - Jimi |
| 5 | SADNESS_04 | 00:53 | 0 % | Child overhears parents arguing | Avoin tarina - Uusperhe |
| 6 | SADNESS_05 | 00:51 | 83 % | Girl feeling sad | Kanelia kainaloon, Tatu ja Patu! |
| 2 | SADNESS_06 | 00:57 | 0 % | Boy missing father | Avoin tarina - Uusperhe |
| 5 | SADNESS_07 | 00:59 | 5 % | Group of friends saying goodbye | Esteitä ja ylityksiä: Muutto |
| 1 | TENDERNESS_01 | 00:32 | 72 % | Friends baking together | Onnelin ja Annelin talvi |
| 6 | TENDERNESS_02 | 00:57 | 85 % | Sisters discussing | Heinähattu ja Vilttitossu |
| 4 | TENDERNESS_03 | 00:54 | 78 % | Man plays flute, discusses with wife | Onnelin, Anneli ja salaperäinen muukalainen - Jakso 5 - Uusi suunnitelma |
| 3 | TENDERNESS_04 | 01:04 | 0 % | Mother decides to quit drinking | Avoin tarina - Jimi |
| 5 | TENDERNESS_05 | 00:36 | 71 % | Girls camping | Me Rosvolat |
| 2 | TENDERNESS_06 | 00:44 | 17 % | Girl and boy chatting | Ihan sama - Jakso 2 - Alin taju |
| 6 | TENDERNESS_07 | 00:47 | 5 % | Mother consolidates daughter | Esteitä ja ylityksiä: Muutto |

**Supplementary Table 3**

*Frequency of missing data for each modality and age group separately.*

|  |  |  | Age group | | | | | | | |
| --- | --- | --- | --- | --- | --- | --- | --- | --- | --- | --- |
|  |  | 8 | 9 | 10 | 11 | 12 | 13 | 14 | 15 | adult |
| ECG | no ECG | 1 | - | 1 | - | 1 | 2 | - | 1 | - |
|  | partly missing | - | - | - | - | - | - | - | - | 1 |
|  | noisy | 1 | 1 | 1 | 2 | 2 | 4 | 1 | - | 3 |
| Eye-tracking | no eye-tracking | - | - | - | 1 | - | 1 | - | - | 3 |
|  | partly missing | - | 1 | 1 | - | - | - | - | 1 | 1 |
|  | bad calibration | 2 | 3 | 1 | 1 | - | - | - | - | 2 |
| Ratings | no ratings | - | - | - | - | - | 1 | - | - | - |
|  | missing two | - | 1 | 2 | 2 | 2 | 1 | - | - | 1 |
|  | missing one | 1 | 7 | 5 | 2 | 2 | 1 | 1 | 1 | 4 |

**Supplementary Table 4**

*Results of the Linear Age Model: emotion intensity*

| Predictors | Est/Beta | SE | 95% CI | t | p |
| --- | --- | --- | --- | --- | --- |
| Intercept | 49.04 | 3.99 | [41.23, 56.86] | 12.29 | <0.001 |
| Age | -0.86 | 0.27 | [-1.39, -0.34] | -3.21 | 0.002 |
| Tenderness | -27.03 | 3.36 | [-33.57, -20.49] | -8.05 | <0.001 |
| Joy | 16.11 | 3.36 | [9.57, 22.65] | 4.80 | >0.001 |
| Anger | 3.95 | 3.36 | [-2.59, 10.49] | 1.18 | 0.240 |
| Sadness | 5.28 | 3.36 | [-1.26, 11.82] | 1.57 | 0.117 |
| Fear | 1.29 | 3.36 | [-5.26, 7.83] | 0.38 | 0.702 |
| Age x Tenderness | 1.20 | 0.23 | [0.75, 1.64] | 5.27 | <0.001 |
| Age x Joy | -0.30 | 0.23 | [-0.74, 0.14] | -1.33 | 0.184 |
| Age x Anger | -0.45 | 0.23 | [-0.89, -0.01] | -2.00 | 0.046 |
| Age x Sadness | 0.03 | 0.23 | [-0.41, 0.47] | 0.14 | 0.893 |
| Age x Fear | -0.68 | 0.23 | [-1.12, -0.24] | -3.00 | 0.003 |

*Note*. β = Estimate based on -scored predictors; SE = standard error; CI = confidence interval.

**Supplementary Table 5**

*Comparison between continuous and* *polynomial age model: ratings*

|  | **npar** | **AIC** | **BIC** | **logLik** | **deviance** | **Chisq** | **df** | **p** |
| --- | --- | --- | --- | --- | --- | --- | --- | --- |
| Rating continuous  age model | 14 | 6083.0 | 6146.9 | -3027.5 | 6055.0 |  |  |  |
| Rating polynomial  age model | 20 | 6090.8 | 6182.2 | -3025.4 | 6050.8 | 4.139 | 6 | 0.658 |

*Note.* npar – number of parameters in the model; AIC – Akaike Information Criterion; BIC – Bayesian Information Criterion; logLik – the log-likelihood of the model.

**Supplementary Table 6**

*Results of the categorical age model: emotion intensity*

| Predictors | Est/Beta | SE | 95% CI | t | p |
| --- | --- | --- | --- | --- | --- |
| Intercept | 29.56 | 2.86 | [24.02, 35.1] | 10.32 | <0.001 |
| 8-9 | 11.81 | 4.08 | [3.91, 19.71] | 2.89 | 0.005 |
| 10-11 | 8.41 | 4.25 | [0.19, 16.62] | 1.98 | 0.050 |
| 12-13 | 13.76 | 4.40 | [5.25, 22.28] | 3.13 | 0.002 |
| 14-15 | 3.32 | 5.21 | [-6.75, 13.39] | 0.64 | 0.526 |
| Tenderness | -0.34 | 2.43 | [-5.01, 4.32] | -0.14 | 0.889 |
| Joy | 10.23 | 2.43 | [5.56, 14.9] | 4.21 | <0.001 |
| Anger | -6.66 | 2.43 | [-11.32, -1.99] | -2.74 | 0.006 |
| Sadness | 6.05 | 2.43 | [1.38, 10.72] | 2.49 | 0.013 |
| Fear | -13.62 | 2.43 | [-18.29, -8.96] | -5.61 | <0.001 |
| 8-9 x Tenderness | -18.12 | 3.46 | [-24.78, -11.47] | -5.23 | <0.001 |
| 10-11 x Tenderness | -14.60 | 3.60 | [-21.52, -7.68] | -4.05 | <0.001 |
| 12-13 x Tenderness | -10.69 | 3.74 | [-17.87, -3.51] | -2.86 | 0.004 |
| 14-15 x Tenderness | -6.41 | 4.42 | [-14.9, 2.07] | -1.45 | 0.147 |
| 8-9 x Joy | 5.41 | 3.46 | [-1.24, 12.07] | 1.56 | 0.119 |
| 10-11 x Joy | 2.25 | 3.60 | [-4.67, 9.17] | 0.63 | 0.532 |
| 12-13 x Joy | 0.00 | 3.74 | [-7.17, 7.18] | 0.00 | 0.999 |
| 14-15 x Joy | -0.66 | 4.42 | [-9.15, 7.82] | -0.15 | 0.880 |
| 8-9 x Anger | 5.39 | 3.46 | [-1.27, 12.04] | 1.56 | 0.120 |
| 10-11 x Anger | 6.82 | 3.60 | [-0.1, 13.74] | 1.89 | 0.059 |
| 12-13 x Anger | 6.64 | 3.74 | [-0.53, 13.82] | 1.78 | 0.076 |
| 14-15 x Anger | 3.32 | 4.42 | [-5.16, 11.81] | 0.75 | 0.452 |
| 8-9 x Sadness | -1.28 | 3.46 | [-7.93, 5.38] | -0.37 | 0.713 |
| 10-11 x Sadness | 1.07 | 3.60 | [-5.85, 7.99] | 0.30 | 0.766 |
| 12-13 x Sadness | -1.47 | 3.74 | [-8.65, 5.7] | -0.39 | 0.694 |
| 14-15 x Sadness | 0.11 | 4.42 | [-8.38, 8.59] | 0.02 | 0.981 |
| 8-9 x Fear | 10.69 | 3.46 | [4.04, 17.35] | 3.09 | 0.002 |
| 10-11 x Fear | 7.26 | 3.60 | [0.34, 14.18] | 2.02 | 0.044 |
| 12-13 x Fear | 4.81 | 3.78 | [-2.46, 12.08] | 1.27 | 0.204 |
| 14-15 x Fear | 4.61 | 4.42 | [-3.88, 13.1] | 1.04 | 0.297 |

*Note*. β = Estimate based on -scored predictors; SE = standard error; CI = confidence interval.

**Supplementary Table 7**

*Estimated marginal means of emotion categories: emotion intensity*

| Category | Emmean | SE | df | Lower.CL | Upper.CL |
| --- | --- | --- | --- | --- | --- |
| Tenderness | 26.6 | 1.88 | 310 | 22.9 | 30.3 |
| Joy | 49.1 | 1.88 | 310 | 45.4 | 52.8 |
| Anger | 34.8 | 1.88 | 310 | 31.1 | 38.5 |
| Sadness | 42.8 | 1.88 | 310 | 39.1 | 46.5 |
| Fear | 29.0 | 1.89 | 313 | 25.3 | 32.7 |
| Amusement | 40.4 | 1.88 | 310 | 36.7 | 44.1 |

*Note*. Emmean = estimated mean

**Supplementary Table 8**

*Pairwise comparisons between emotion categories: emotion intensity*

| Contrast | Estimate | SE | df | t.ratio | p |
| --- | --- | --- | --- | --- | --- |
| Tenderness - Joy | -22.45 | 1.88 | 584 | -11.96 | <0.001 |
| Tenderness - Anger | -8.18 | 1.88 | 584 | -4.36 | <0.001 |
| Tenderness - Sadness | -16.21 | 1.88 | 584 | -8.63 | <0.001 |
| Tenderness - Fear | -2.40 | 1.88 | 584 | -1.28 | 0.797 |
| Tenderness - Amusement | -13.77 | 1.88 | 584 | -7.34 | <0.001 |
| Joy - Anger | 14.27 | 1.88 | 584 | 7.60 | <0.001 |
| Joy - Sadness | 6.25 | 1.88 | 584 | 3.34 | 0.012 |
| Joy - Fear | 20.05 | 1.88 | 584 | 10.65 | <0.001 |
| Joy - Amusement | 8.68 | 1.88 | 584 | 4.63 | <0.001 |
| Anger - Sadness | -8.02 | 1.88 | 584 | -4.27 | <0.001 |
| Anger - Fear | 5.78 | 1.88 | 584 | 3.07 | 0.0270 |
| Anger - Amusement | -5.59 | 1.88 | 584 | -2.98 | 0.0358 |
| Sadness - Fear | 13.80 | 1.88 | 584 | 7.33 | <0.001 |
| Sadness - Amusement | 2.44 | 1.88 | 584 | 1.30 | 0.787 |
| Fear - Amusement | -11.37 | 1.88 | 584 | -6.04 | <0.001 |

*Note*. Tukey's HSD method was used to correct for multiple comparisons.

**Supplementary Table 9**

*Results of the curvilinear age model: HR*

| Predictors | Est/Beta | SE | 95% CI | t | p |
| --- | --- | --- | --- | --- | --- |
| (Intercept) | 115.55 | 7.71 | [100.51, 130.6] | 14.98 | <0.001 |
| Age | -4.82 | 1.04 | [-6.86, -2.78] | -4.61 | <0.001 |
| Age^2^ | 0.14 | 0.03 | [0.08, 0.2] | 4.36 | <0.001 |
| Tenderness | 0.70 | 0.84 | [-0.93, 2.33] | 0.83 | 0.404 |
| Joy | -0.59 | 0.84 | [-2.22, 1.04] | -0.70 | 0.483 |
| Anger | 1.48 | 0.84 | [-0.15, 3.11] | 1.76 | 0.079 |
| Sadness | -1.61 | 0.84 | [-3.24, 0.02] | -1.92 | 0.056 |
| Fear | 0.43 | 0.84 | [-1.2, 2.06] | 0.51 | 0.609 |
| Age x Tenderness | -0.12 | 0.11 | [-0.34, 0.1] | -1.02 | 0.307 |
| Age x Joy | 0.11 | 0.11 | [-0.11, 0.34] | 1.01 | 0.313 |
| Age x Anger | -0.18 | 0.11 | [-0.4, 0.04] | -1.58 | 0.115 |
| Age x Sadness | 0.17 | 0.11 | [-0.05, 0.4] | 1.54 | 0.125 |
| Age x Fear | 0.08 | 0.11 | [-0.14, 0.3] | 0.74 | 0.459 |
| Age^2^ x Tenderness | 0.00 | 0.00 | [0, 0.01] | 1.17 | 0.241 |
| Age^2^ x Joy | 0.00 | 0.00 | [-0.01, 0] | -1.14 | 0.255 |
| Age^2^ x Anger | 0.00 | 0.00 | [0, 0.01] | 1.21 | 0.227 |
| Age^2^ x Sadness | 0.00 | 0.00 | [-0.01, 0] | -1.38 | 0.169 |
| Age^2^ x Fear | 0.00 | 0.00 | [-0.01, 0] | -0.92 | 0.360 |

*Note*. β = Estimate based on -scored predictors; SE = standard error; CI = confidence interval.

**Supplementary Table 10**

*Comparison between continuous and* *polynomial age model: HR*

|  | npar | AIC | BIC | logLik | deviance | Chisq | df | p |
| --- | --- | --- | --- | --- | --- | --- | --- | --- |
| HR continuous  age model | 14 | 2743.4 | 2806.8 | -1357.7 | 2715.4 |  |  |  |
| HR polynomial  age model | 20 | 2730.6 | 2821.1 | -1345.3 | 2690.6 | 24.83 | 6 | <0.001 |

*Note.* npar – number of parameters in the model; AIC – Akaike Information Criterion; BIC – Bayesian Information Criterion; logLik – the log-likelihood of the model

**Supplementary Table 11**

*Results of the categorical age model: HR*

| Predictors | Est/Beta | SE | 95% CI | t | p |
| --- | --- | --- | --- | --- | --- |
| Intercept | 77.73 | 1.68 | [74.49, 80.97] | 46.37 | <0.001 |
| 8-9 | 6.65 | 2.41 | [1.99, 11.31] | 2.76 | 0.007 |
| 10-11 | 1.49 | 2.51 | [-3.37, 6.35] | 0.59 | 0.556 |
| 12-13 | -0.24 | 2.65 | [-5.37, 4.88] | -0.09 | 0.927 |
| 14-15 | -5.02 | 3.14 | [-11.08, 1.04] | -1.60 | 0.113 |
| Tenderness | 0.13 | 0.18 | [-0.21, 0.47] | 0.72 | 0.470 |
| Joy | 0.06 | 0.18 | [-0.28, 0.4] | 0.34 | 0.733 |
| Anger | -0.52 | 0.18 | [-0.86, -0.18] | -2.92 | 0.004 |
| Sadness | -0.16 | 0.18 | [-0.51, 0.18] | -0.93 | 0.355 |
| Fear | 0.79 | 0.18 | [0.45, 1.13] | 4.46 | <0.001 |
| 8-9 x Tenderness | -0.11 | 0.26 | [-0.6, 0.38] | -0.42 | 0.672 |
| 10-11 x Tenderness | -0.18 | 0.27 | [-0.69, 0.33] | -0.69 | 0.493 |
| 12-13 x Tenderness | -0.40 | 0.28 | [-0.93, 0.14] | -1.41 | 0.159 |
| 14-15 x Tenderness | -0.15 | 0.33 | [-0.78, 0.49] | -0.44 | 0.657 |
| 8-9 x Joy | 0.03 | 0.26 | [-0.46, 0.52] | 0.12 | 0.908 |
| 10-11 x Joy | 0.31 | 0.27 | [-0.2, 0.82] | 1.17 | 0.241 |
| 12-13 x Joy | 0.06 | 0.28 | [-0.48, 0.6] | 0.22 | 0.828 |
| 14-15 x Joy | -0.04 | 0.33 | [-0.68, 0.6] | -0.12 | 0.905 |
| 8-9 x Anger | 0.76 | 0.26 | [0.27, 1.25] | 2.96 | 0.003 |
| 10-11 x Anger | 0.31 | 0.27 | [-0.2, 0.82] | 1.17 | 0.242 |
| 12-13 x Anger | 0.59 | 0.28 | [0.05, 1.13] | 2.11 | 0.035 |
| 14-15 x Anger | 0.59 | 0.33 | [-0.05, 1.23] | 1.78 | 0.076 |
| 8-9 x Sadness | -0.43 | 0.26 | [-0.92, 0.06] | -1.69 | 0.092 |
| 10-11 x Sadness | 0.10 | 0.27 | [-0.41, 0.61] | 0.37 | 0.708 |
| 12-13 x Sadness | 0.00 | 0.28 | [-0.54, 0.54] | 0.01 | 0.995 |
| 14-15 x Sadness | 0.17 | 0.33 | [-0.47, 0.8] | 0.50 | 0.614 |
| 8-9 x Fear | 0.22 | 0.26 | [-0.27, 0.71] | 0.85 | 0.394 |
| 10-11 x Fear | 0.07 | 0.27 | [-0.44, 0.58] | 0.26 | 0.795 |
| 12-13 x Fear | 0.10 | 0.28 | [-0.44, 0.64] | 0.35 | 0.727 |
| 14-15 x Fear | 0.26 | 0.33 | [-0.37, 0.9] | 0.79 | 0.427 |

*Note*. β = Estimate based on -scored predictors; SE = standard error; CI = confidence interval.

**Supplementary Table 12**

*Estimated marginal means of emotion categories: HR*

| Category | EMMEAN | SE | df | Lower.CL | Upper.CL |
| --- | --- | --- | --- | --- | --- |
| Tenderness | 79.1 | 0.836 | 114 | 77.4 | 80.7 |
| Joy | 79.2 | 0.836 | 114 | 77.6 | 80.9 |
| Anger | 79.0 | 0.836 | 114 | 77.4 | 80.7 |
| Sadness | 78.9 | 0.836 | 114 | 77.2 | 80.5 |
| Fear | 80.0 | 0.836 | 114 | 78.4 | 81.7 |
| Amusement | 78.4 | 0.836 | 114 | 76.8 | 80.1 |

**Supplementary Table 13**

*Pairwise comparisons between emotion categories: HR*

| Contrast | Estimate | SE | df | t.ratio | p |
| --- | --- | --- | --- | --- | --- |
| Tenderness - Joy | -0.1618 | 0.14 | 555 | -1.15 | 0.859 |
| Tenderness - Anger | 0.0781 | 0.14 | 555 | 0.56 | 0.994 |
| Tenderness - Sadness | 0.2096 | 0.14 | 555 | 1.49 | 0.668 |
| Tenderness - Fear | -0.9256 | 0.14 | 555 | -6.60 | <0.001 |
| Tenderness - Amusement | 0.6691 | 0.14 | 555 | 4.77 | <0.001 |
| Joy - Anger | 0.2399 | 0.14 | 555 | 1.71 | 0.526 |
| Joy - Sadness | 0.3714 | 0.14 | 555 | 2.65 | 0.088 |
| Joy - Fear | -0.7638 | 0.14 | 555 | -5.44 | <0.001 |
| Joy - Amusement | 0.8309 | 0.14 | 555 | 5.92 | <0.001 |
| Anger - Sadness | 0.1315 | 0.14 | 555 | 0.94 | 0.937 |
| Anger - Fear | -1.0037 | 0.14 | 555 | -7.15 | <0.001 |
| Anger - Amusement | 0.5909 | 0.14 | 555 | 4.21 | <0.001 |
| Sadness - Fear | -1.1352 | 0.14 | 555 | -8.09 | <0.001 |
| Sadness - Amusement | 0.4595 | 0.14 | 555 | 3.26 | 0.014 |
| Fear - Amusement | 1.5946 | 0.14 | 555 | 11.37 | <0.001 |

*Note*. Tukey's HSD method was used to correct for multiple comparisons.

**Supplementary Table 14**

*Results of the continuous age model: HRV*

| Predictors | Est/Beta | SE | 95% CI | t | p |
| --- | --- | --- | --- | --- | --- |
| Intercept | 77.68 | 6.00 | [65.92, 89.45] | 12.94 | <0.001 |
| Age | -1.66 | 0.40 | [-2.44, -0.87] | -4.12 | <0.001 |
| Tenderness | 1.31 | 1.19 | [-1, 3.62] | 1.10 | 0.271 |
| Joy | -1.25 | 1.19 | [-3.56, 1.06] | -1.05 | 0.292 |
| Anger | -0.69 | 1.19 | [-3, 1.62] | -0.58 | 0.560 |
| Sadness | 1.92 | 1.19 | [-0.39, 4.23] | 1.62 | 0.107 |
| Fear | -0.16 | 1.19 | [-2.47, 2.15] | -0.14 | 0.891 |
| Age x Tenderness | -0.04 | 0.08 | [-0.19, 0.12] | -0.46 | 0.643 |
| Age x Joy | 0.04 | 0.08 | [-0.11, 0.2] | 0.56 | 0.578 |
| Age x Anger | 0.08 | 0.08 | [-0.07, 0.23] | 1.01 | 0.313 |
| Age x Sadness | -0.15 | 0.08 | [-0.31, 0] | -1.95 | 0.052 |
| Age x Fear | -0.03 | 0.08 | [-0.18, 0.12] | -0.38 | 0.703 |

*Note*. β = Estimate based on -scored predictors; SE = standard error; CI = confidence interval.

**Supplementary Table 15**

*Comparison between continuous and polynomial age models: HRV*

|  | npar | AIC | BIC | logLik | deviance | Chisq | df | p |
| --- | --- | --- | --- | --- | --- | --- | --- | --- |
| HRV continuous  age model | 14 | 4682.1 | 4745.3 | -2327.0 | 4654.1 |  |  |  |
| HRV polynomial  age model | 20 | 4686.9 | 4777.3 | -2323.4 | 4646.9 | 7.177 | 6 | 0.305 |

*Note.* npar – number of parameters in the model; AIC – Akaike Information Criterion; BIC – Bayesian Information Criterion; logLik – the log-likelihood of the model.

**Supplementary Table 16**

*Results of the categorical age model: HRV*

| Predictors | Est/Beta | SE | 95% CI | t | p |
| --- | --- | --- | --- | --- | --- |
| Intercept | 38.62 | 3.77 | [31.34, 45.91] | 10.25 | <0.001 |
| 8-9 | 16.28 | 5.48 | [5.7, 26.86] | 2.97 | 0.004 |
| 10-11 | 22.36 | 5.72 | [11.31, 33.43] | 3.91 | <0.001 |
| 12-13 | 21.12 | 5.96 | [9.61, 32.63] | 3.54 | <0.001 |
| 14-15 | 19.48 | 7.05 | [5.86, 33.1] | 2.76 | 0.007 |
| Tenderness | 0.97 | 0.80 | [-0.56, 2.5] | 1.22 | 0.223 |
| Joy | -0.37 | 0.80 | [-1.9, 1.16] | -0.46 | 0.643 |
| Anger | 0.86 | 0.80 | [-0.67, 2.39] | 1.08 | 0.280 |
| Sadness | -1.15 | 0.80 | [-2.68, 0.37] | -1.45 | 0.148 |
| Fear | -1.19 | 0.80 | [-2.72, 0.33] | -1.50 | 0.135 |
| 8-9 x Tenderness | 0.10 | 1.17 | [-2.14, 2.34] | 0.08 | 0.934 |
| 10-11 x Tenderness | 0.27 | 1.22 | [-2.08, 2.62] | 0.22 | 0.825 |
| 12-13 x Tenderness | -1.50 | 1.26 | [-3.92, 0.92] | -1.19 | 0.234 |
| 14-15 x Tenderness | -0.70 | 1.49 | [-3.56, 2.16] | -0.47 | 0.639 |
| 8-9 x Joy | -1.52 | 1.17 | [-3.77, 0.72] | -1.30 | 0.193 |
| 10-11 x Joy | -0.13 | 1.22 | [-2.48, 2.22] | -0.11 | 0.914 |
| 12-13 x Joy | 2.18 | 1.26 | [-0.24, 4.6] | 1.73 | 0.084 |
| 14-15 x Joy | -1.77 | 1.49 | [-4.62, 1.09] | -1.18 | 0.237 |
| 8-9 x Anger | -1.54 | 1.17 | [-3.78, 0.71] | -1.31 | 0.189 |
| 10-11 x Anger | -0.39 | 1.22 | [-2.74, 1.95] | -0.32 | 0.747 |
| 12-13 x Anger | 0.11 | 1.26 | [-2.31, 2.53] | 0.09 | 0.931 |
| 14-15 x Anger | -0.78 | 1.49 | [-3.64, 2.08] | -0.52 | 0.601 |
| 8-9 x Sadness | 3.09 | 1.17 | [0.84, 5.33] | 2.64 | 0.009 |
| 10-11 x Sadness | -0.11 | 1.22 | [-2.46, 2.23] | -0.09 | 0.926 |
| 12-13 x Sadness | -1.02 | 1.28 | [-3.47, 1.43] | -0.80 | 0.426 |
| 14-15 x Sadness | 1.41 | 1.49 | [-1.44, 4.27] | 0.95 | 0.343 |
| 8-9 x Fear | 0.53 | 1.17 | [-1.71, 2.77] | 0.45 | 0.650 |
| 10-11 x Fear | 1.63 | 1.22 | [-0.72, 3.98] | 1.33 | 0.184 |
| 12-13 x Fear | 1.29 | 1.26 | [-1.13, 3.71] | 1.02 | 0.306 |
| 14-15 x Fear | 1.03 | 1.49 | [-1.83, 3.89] | 0.69 | 0.489 |

*Note*. β = Estimate based on -scored predictors; SE = standard error; CI = confidence interval.

**Supplementary Table 17**

*Continuous age model: gaze behaviour*

| Predictors | Est/Beta | SE | 95% CI | t | p |
| --- | --- | --- | --- | --- | --- |
| Intercept | 84.22 | 1.99 | [80.32, 88.12] | 42.28 | <0.001 |
| Age | 0.26 | 0.14 | [0, 0.53] | 1.93 | 0.056 |
| Tenderness | -2.32 | 0.71 | [-3.69, -0.94] | -3.27 | 0.001 |
| Joy | 2.36 | 0.71 | [0.99, 3.74] | 3.34 | <0.001 |
| Anger | 2.77 | 0.71 | [1.39, 4.15] | 3.91 | <0.001 |
| Sadness | -1.73 | 0.71 | [-3.11, -0.35] | -2.45 | 0.015 |
| Fear | -6.06 | 0.71 | [-7.44, -4.68] | -8.57 | <0.001 |
| Age x Tenderness | 0.02 | 0.05 | [-0.08, 0.11] | 0.32 | 0.750 |
| Age x Joy | -0.01 | 0.05 | [-0.1, 0.08] | -0.20 | 0.845 |
| Age x Anger | -0.05 | 0.05 | [-0.15, 0.04] | -1.07 | 0.285 |
| Age x Sadness | -0.02 | 0.05 | [-0.11, 0.08] | -0.32 | 0.749 |
| Age x Fear | 0.21 | 0.05 | [0.12, 0.31] | 4.40 | <0.001 |

*Note*. β = Estimate based on -scored predictors; SE = standard error; CI = confidence interval.

**Supplementary Table 18**

*Comparison between continues and polynomial age model: gaze behaviour*

|  | npar | AIC | BIC | logLik | deviance | Chisq | df | p |
| --- | --- | --- | --- | --- | --- | --- | --- | --- |
| Gaze continues  age model | 14 | 3900.9 | 3964.4 | -1936.5 | 3872.9 |  |  |  |
| Gaze polynomial  age model | 20 | 3905.8 | 3996.5 | -1932.9 | 3865.8 | 7.068 | 6 | 0.315 |

*Note.* npar – number of parameters in the model; AIC – Akaike Information Criterion; BIC – Bayesian Information Criterion; logLik – the log-likelihood of the model.

**Supplementary Table 19**

*Categorical age model: gaze behaviour*

| Predictors | Est/Beta | SE | 95% CI | t | p |
| --- | --- | --- | --- | --- | --- |
| (Intercept) | 89.47 | 1.45 | [86.67, 92.27] | 61.69 | <0.001 |
| 8-9 | -5.43 | 2.02 | [-9.32, -1.53] | -2.69 | 0.008 |
| 10-11 | -0.84 | 2.11 | [-4.93, 3.25] | -0.40 | 0.692 |
| 12-13 | 0.60 | 2.16 | [-3.58, 4.78] | 0.28 | 0.782 |
| 14-15 | -2.03 | 2.54 | [-6.95, 2.89] | -0.80 | 0.427 |
| Tenderness | -2.28 | 0.53 | [-3.29, -1.27] | -4.33 | <0.001 |
| Joy | 1.86 | 0.53 | [0.85, 2.87] | 3.53 | <0.001 |
| Anger | 1.87 | 0.53 | [0.86, 2.88] | 3.56 | <0.001 |
| Sadness | -2.01 | 0.53 | [-3.02, -1] | -3.83 | <0.001 |
| Fear | -1.22 | 0.53 | [-2.23, -0.21] | -2.31 | 0.021 |
| 8-9 x Tenderness | 0.09 | 0.73 | [-1.32, 1.49] | 0.12 | 0.907 |
| 10-11 x Tenderness | -0.04 | 0.77 | [-1.51, 1.43] | -0.05 | 0.960 |
| 12-13 x Tenderness | -0.01 | 0.79 | [-1.52, 1.5] | -0.02 | 0.987 |
| 14-15 x Tenderness | 1.41 | 0.92 | [-0.36, 3.18] | 1.53 | 0.128 |
| 8-9 x Joy | 0.36 | 0.73 | [-1.05, 1.76] | 0.49 | 0.624 |
| 10-11 x Joy | 0.24 | 0.77 | [-1.24, 1.71] | 0.31 | 0.758 |
| 12-13 x Joy | 0.44 | 0.79 | [-1.07, 1.95] | 0.56 | 0.576 |
| 14-15 x Joy | 1.34 | 0.92 | [-0.43, 3.12] | 1.46 | 0.146 |
| 8-9 x Anger | -0.04 | 0.73 | [-1.45, 1.36] | -0.06 | 0.952 |
| 10-11 x Anger | 1.13 | 0.77 | [-0.34, 2.61] | 1.48 | 0.140 |
| 12-13 x Anger | 0.60 | 0.79 | [-0.91, 2.11] | 0.76 | 0.446 |
| 14-15 x Anger | -1.32 | 0.92 | [-3.09, 0.45] | -1.43 | 0.153 |
| 8-9 x Sadness | 0.10 | 0.73 | [-1.3, 1.5] | 0.14 | 0.891 |
| 10-11 x Sadness | 0.27 | 0.77 | [-1.2, 1.74] | 0.35 | 0.726 |
| 12-13 x Sadness | -0.11 | 0.79 | [-1.62, 1.4] | -0.14 | 0.890 |
| 14-15 x Sadness | 0.07 | 0.92 | [-1.7, 1.84] | 0.08 | 0.939 |
| 8-9 x Fear | -2.82 | 0.73 | [-4.23, -1.42] | -3.86 | <0.001 |
| 10-11 x Fear | -2.95 | 0.77 | [-4.42, -1.48] | -3.85 | <0.001 |
| 12-13 x Fear | -1.85 | 0.79 | [-3.36, -0.34] | -2.35 | 0.019 |
| 14-15 x Fear | -2.27 | 0.92 | [-4.04, -0.5] | -2.46 | 0.014 |

*Note*. β = Estimate based on -scored predictors; SE = standard error; CI = confidence interval.

**Supplementary Table 20**

*Estimated marginal means of emotion categories: gaze behaviour*

| Category | EM Mean | SE | df | Lower CL | Upper CL |
| --- | --- | --- | --- | --- | --- |
| Tenderness | 85.7 | 0.759 | 143 | 84.2 | 87.2 |
| Joy | 90.0 | 0.759 | 143 | 88.5 | 91.5 |
| Anger | 89.9 | 0.759 | 143 | 88.4 | 91.4 |
| Sadness | 85.9 | 0.759 | 143 | 84.4 | 87.4 |
| Fear | 84.7 | 0.759 | 143 | 83.2 | 86.2 |
| Amusement | 90.7 | 0.760 | 143 | 89.2 | 92.2 |

**Supplementary Table 21**

*Pairwise comparisons between emotion categories: gaze behaviour*

| Contrast | Estimate | SE | df | t.ratio | p |
| --- | --- | --- | --- | --- | --- |
| Tenderness - Joy | -4.340 | 0.394 | 564 | -11.027 | <0.001 |
| Tenderness - Anger | -4.168 | 0.394 | 564 | -10.591 | <0.001 |
| Tenderness - Sadness | -0.162 | 0.394 | 564 | -0.411 | 0.999 |
| Tenderness - Fear | 1.049 | 0.394 | 564 | 2.665 | 0.084 |
| Tenderness - Amusement | -5.009 | 0.395 | 564 | -12.692 | <0.001 |
| Joy - Anger | 0.172 | 0.394 | 564 | 0.436 | 0.998 |
| Joy - Sadness | 4.178 | 0.394 | 564 | 10.616 | <0.001 |
| Joy - Fear | 5.389 | 0.394 | 564 | 13.692 | <0.001 |
| Joy - Amusement | -0.669 | 0.395 | 564 | -1.694 | 0.536 |
| Anger - Sadness | 4.007 | 0.394 | 564 | 10.180 | <0.001 |
| Anger - Fear | 5.217 | 0.394 | 564 | 13.256 | <0.001 |
| Anger - Amusement | -0.840 | 0.395 | 564 | -2.129 | 0.274 |
| Sadness - Fear | 1.211 | 0.394 | 564 | 3.076 | 0.027 |
| Sadness - Amusement | -4.847 | 0.395 | 564 | -12.283 | <0.001 |
| Fear - Amusement | -6.058 | 0.395 | 564 | -15.350 | <0.001 |

*Note*. Tukey's HSD method was used to correct for multiple comparisons.

## Supplementary analyses

### Linear mixed-effects models for non-target emotion

We ran an additional analysis to account for non-target emotions using LMM by calculating the average intensity of mixed emotions. In this approach, we divided the sum of non-target ratings by the number of non-zero non-target ratings to calculate the average intensity per non-target emotion. This method balances the intensity of mixed emotions with the number of emotions reported, making comparisons straightforward. It helps in understanding whether participants tend to experience mild or strong mixed emotions on average, regardless of how many non-target emotions they report.

When studying developmental trends (e.g., how mixed emotions change with age), averaging provides a consistent way to compare age groups. In our case, younger children might report fewer emotions with higher intensities, while older participants report many but subtler emotions. The average metric reflects these patterns more accurately than the raw sum (which we also ran in comparison). We then included this metric in our standard linear mixed-effects models. The results are shown below in Figure 2 and Tables 22-25.

Analysis showed that participants reported mixed emotions depending on the emotional category of the clip. For instance, during joy-inducing clips, participants more frequently reported high-intensity non-target emotions, whereas mixed emotions were less commonly reported during fear-inducing clips. The average intensity of non-target emotions decreased with age, mirroring the trend observed in the intensity of target emotions.

**Supplementary Figure 2**

*Average intensity per non-target emotion for each clip category separately. For visualization purposes, we used the model with age as a categorical variable.*


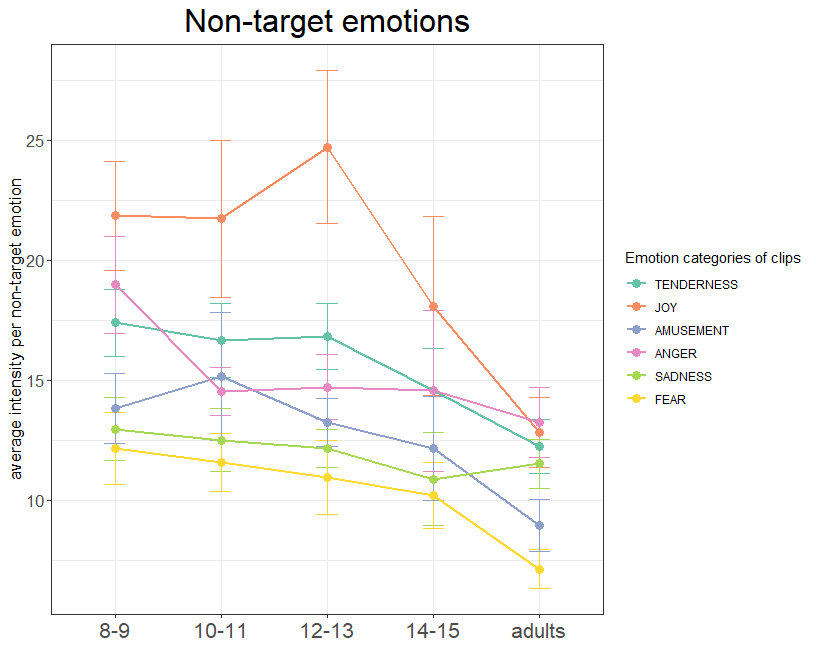


**Supplementary Table 22**

*Effects of age group and category on non-target emotions intensity (ANOVA)*

| Predictors | Sum Sq | Mean Sq | NumDF | DenDF | F value | p |
| --- | --- | --- | --- | --- | --- | --- |
| Age Group | 595.40 | 148.86 | 4 | 113.24 | 3.33 | 0.013 |
| Category | 6152.10 | 1230.42 | 5 | 562.09 | 27.53 | <0.001 |
| Age Group x Category | 1408.60 | 70.43 | 20 | 562.14 | 1.58 | 0.053 |

**Supplementary Table 23**

*Results of the categorical age model: non-target emotions intensity*

| Predictors | Est/Beta | SE | df | t | p |
| --- | --- | --- | --- | --- | --- |
| Intercept | 10.99 | 1.11 | 112.55 | 9.94 | <0.001 |
| Age Group 8-9 | 5.18 | 1.58 | 113.21 | 3.28 | 0.001 |
| Age Group 10-11 | 4.38 | 1.64 | 112.55 | 2.67 | 0.009 |
| Age Group 12-13 | 4.28 | 1.71 | 113.83 | 2.51 | 0.013 |
| Age Group 14-15 | 2.43 | 2.01 | 112.55 | 1.21 | 0.230 |
| Category TENDERNESS | 1.26 | 1.11 | 561.46 | 1.13 | 0.259 |
| Category JOY | 1.83 | 1.11 | 561.46 | 1.64 | 0.102 |
| Category ANGER | 2.25 | 1.11 | 561.46 | 2.02 | 0.044 |
| Category SADNESS | 0.54 | 1.11 | 561.46 | 0.49 | 0.627 |
| Category FEAR | -3.85 | 1.11 | 561.46 | -3.46 | <0.001 |
| Age Group 8-9 x TENDERNESS | -0.03 | 1.59 | 561.68 | -0.02 | 0.985 |
| Age Group 10-11 x TENDERNESS | 0.03 | 1.65 | 561.46 | 0.02 | 0.986 |
| Age Group 12-13 x TENDERNESS | 0.29 | 1.72 | 564.00 | 0.17 | 0.865 |
| Age Group 14-15 x TENDERNESS | -0.09 | 2.03 | 561.46 | -0.04 | 0.965 |
| Age Group 8-9 x JOY | 4.18 | 1.62 | 563.25 | 2.58 | 0.010 |
| Age Group 10-11 x JOY | 4.55 | 1.65 | 561.46 | 2.75 | 0.006 |
| Age Group 12-13 x JOY | 7.38 | 1.74 | 562.09 | 4.25 | <0.001 |
| Age Group 14-15 x JOY | 2.86 | 2.03 | 561.46 | 1.41 | 0.159 |
| Age Group 8-9 x ANGER | 0.55 | 1.59 | 561.68 | 0.35 | 0.728 |
| Age Group 10-11 x ANGER | -3.07 | 1.65 | 561.46 | -1.86 | 0.064 |
| Age Group 12-13 x ANGER | -2.81 | 1.72 | 564.00 | -1.63 | 0.103 |
| Age Group 14-15 x ANGER | -1.10 | 2.03 | 561.46 | -0.54 | 0.587 |
| Age Group 8-9 x SADNESS | -3.74 | 1.59 | 561.68 | -2.35 | 0.019 |
| Age Group 10-11 x SADNESS | -3.39 | 1.65 | 561.46 | -2.05 | 0.040 |
| Age Group 12-13 x SADNESS | -3.91 | 1.74 | 562.09 | -2.25 | 0.025 |
| Age Group 14-15 x SADNESS | -3.07 | 2.03 | 561.46 | -1.52 | 0.130 |
| Age Group 8-9 x FEAR | -0.41 | 1.60 | 562.24 | -0.25 | 0.800 |
| Age Group 10-11 x FEAR | 0.05 | 1.65 | 561.46 | 0.03 | 0.974 |
| Age Group 12-13 x FEAR | -0.72 | 1.74 | 562.09 | -0.41 | 0.679 |
| Age Group 14-15 x FEAR | 0.63 | 2.03 | 561.46 | 0.31 | 0.754 |

**Supplementary Table 24**

*Effects of age and category on non-target emotions intensity (ANOVA)*

| Predictors | Sum Sq | Mean Sq | NumDF | DenDF | F value | p |
| --- | --- | --- | --- | --- | --- | --- |
| Age | 618.74 | 618.74 | 1 | 115.88 | 13.91 | <0.001 |
| Category | 2715.45 | 543.09 | 5 | 577.11 | 12.21 | <0.001 |
| Age x Category | 841.41 | 168.28 | 5 | 576.81 | 3.78 | 0.002 |

**Supplementary Table 25**

*Results of the continuous age model: non-target emotions intensity*

| Predictors | Est/Beta | SE | 95% CI | t | p |
| --- | --- | --- | --- | --- | --- |
| Intercept | 19.52 | 1.52 | [16.52, 22.52] | 12.82 | <0.001 |
| Age | -0.38 | 0.10 | [-0.58, -0.19] | -3.73 | <0.001 |
| Tenderness | 1.28 | 1.55 | [-1.77, 4.33] | 0.82 | 0.410 |
| Joy | 10.88 | 1.57 | [7.80, 13.96] | 6.92 | <0.001 |
| Anger | -0.20 | 1.55 | [-3.25, 2.85] | -0.13 | 0.897 |
| Sadness | -6.21 | 1.55 | [-9.26, -3.16] | -4.00 | <0.001 |
| Fear | -4.44 | 1.56 | [-7.51, -1.37] | -2.85 | 0.005 |
| Age x Tenderness | 0.00 | 0.10 | [-0.20, 0.20] | 0.02 | 0.987 |
| Age x Joy | -0.39 | 0.11 | [-0.60, -0.19] | -3.71 | <0.001 |
| Age x Anger | 0.09 | 0.10 | [-0.12, 0.31] | 0.90 | 0.367 |
| Age x Sadness | 0.29 | 0.10 | [0.09, 0.50] | 2.82 | 0.005 |
| Age x Fear | 0.03 | 0.10 | [-0.18, 0.25] | 0.30 | 0.763 |

### Linear mixed-effects models for experienced emotions

In the main analysis, we examined the physiological and behavioural reactions in response to predetermined categories (the "target emotions" approach), where we used a pre-selected set of video clips representing specific emotional categories. These categories were determined based on the ratings of an independent sample of adults. In this approach, the emotional categorization of the clips was considered the "ground truth", presupposing their effectiveness in eliciting the intended emotions across all participants.

The second perspective, the "experienced emotions" approach, which we are reporting here, utilized the same set of video clips. However, rather than assuming a universal emotional response, we relied on each participant's subjective evaluations of their emotional experiences. From these evaluations, a personalized list of clips was generated for each participant, representing six emotions: amusement, anger, fear, joy, tenderness, and sadness. When a participant reported multiple emotions for a single clip, the clip was categorized based on the dominant, most intense emotion. Consequently, this approach allowed for the possibility that a clip categorized under a specific "target emotion" (e.g., fear) could be perceived differently (e.g., as anger or amusement) in the "experienced emotions" analysis.

In both methodologies, the same six emotional categories were used, offering a comparison between the perceived and intended emotional impacts of the video clips. The rationale for this comparison stems from variations in individual responses, which can be observed on the heatmaps illustrating the correspondence between the "target emotions" and "experienced emotions" approaches (Figure 1).

For the analyses, we used the same three-model approach as in the main analysis. Here we are reporting the results of categorical age model, where age was treated as a categorical variable, with distinct age groups compared to adults (reference group).

**Supplementary Figure 3**

*Responses by age group to six experienced emotions*

*
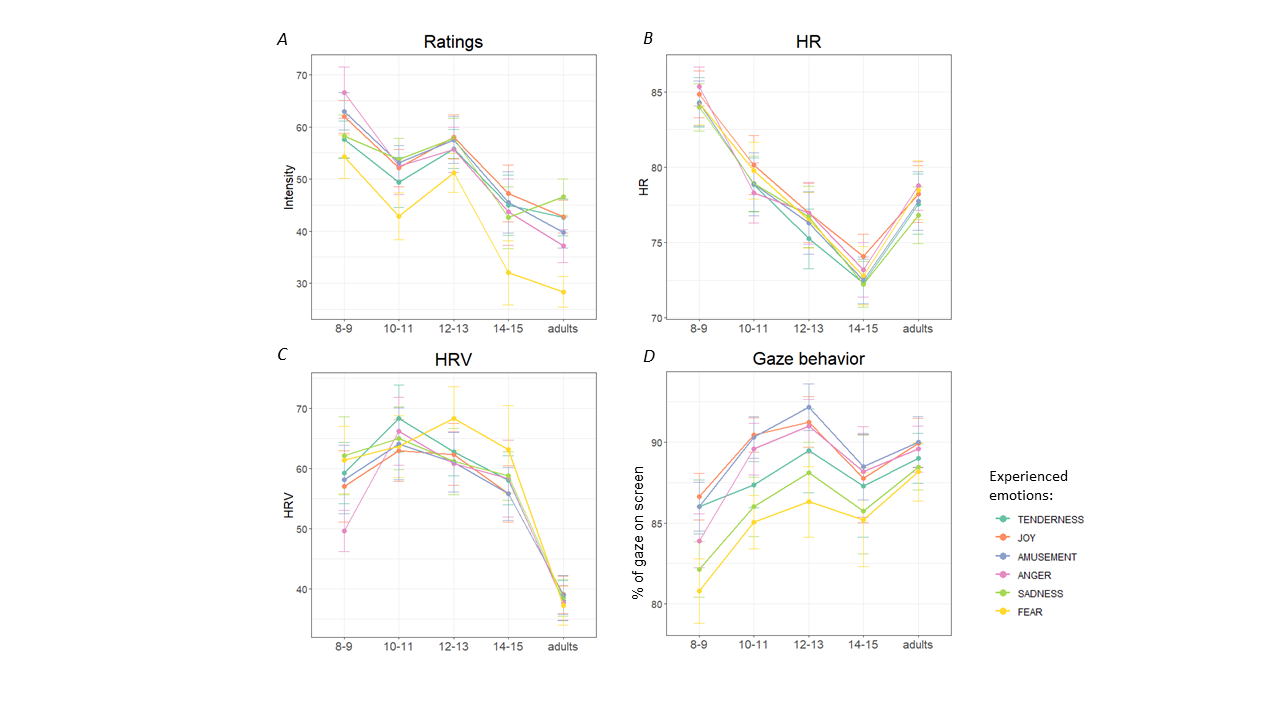
Note.* **A**. Ratings depict the intensity of the experienced emotion as rated by participants. **B.-C.** Heart Rate (HR) and Heart rate Variability (HRV): Indicate physiological responses to emotional stimuli. **D.** Differences in gaze behaviour: Measured as the percentage of time participants looked on the screen

When analysing experienced emotions, we observed similar trends to those seen with targeted emotional categories, although the effects were often weaker (Sup. Figure 3, Sup. Tables 26-33). The results indicate significant age-related variations in emotional intensity. Younger age groups (8-13 years) displayed higher intensity than adults, but the 14–15-year age group did not differ significantly from adults. Emotional intensity also varied depending on the type of emotion experienced; sadness was more intense than average, while fear was the least intense. The HR model revealed that participants aged 8-9 years had a higher heart rate (HR). The experience of joy and fear elicited a higher HR compared to the grand mean, while the experience of sadness led to a lower HR. We also found age-related variations in heart rate variability (HRV), with children (8-15 years) exhibiting higher HRV than adults. However, the effects for experienced emotion and the interaction between age and experienced emotion were not significant. Lastly, participants aged 8-9 years showed increased avoidance behaviour, but no significant differences were observed for other age groups or experienced emotions.

**Supplementary Table 26**

*Effects of age group and category on emotion intensity (ANOVA)*

| Factor | SS | MS | df | F | p |
| --- | --- | --- | --- | --- | --- |
| Age group | 2376.1 | 594.03 | 4 | 5.8738 | <0.001 |
| Category | 9286.5 | 1857.31 | 5 | 18.3651 | <0.001 |
| Age group x Category | 3283.0 | 164.15 | 20 | 1.6231 | 0.043 |

**Supplementary Table 27**

*Results of the categorical age model: emotion intensity*

| Predictors | Est/Beta | SE | t | p |
| --- | --- | --- | --- | --- |
| (Intercept) | 39.80 | 3.30 | 12.05 | <0.001 |
| 8-9 | 20.31 | 4.71 | 4.31 | <0.001 |
| 10-11 | 10.96 | 4.90 | 2.24 | 0.027 |
| 12-13 | 15.45 | 5.08 | 3.04 | 0.003 |
| 14-15 | 1.47 | 6.01 | 0.25 | 0.807 |
| Tenderness | 2.88 | 1.68 | 1.71 | 0.087 |
| Joy | 2.95 | 1.68 | 1.76 | 0.080 |
| Anger | -0.93 | 1.74 | -0.53 | 0.594 |
| Sadness | 6.74 | 1.68 | 4.02 | <0.001 |
| Fear | -11.63 | 1.71 | -6.81 | <0.001 |
| 8-9 x Tenderness | -5.62 | 2.52 | -2.23 | 0.026 |
| 10-11 x Tenderness | -3.78 | 2.55 | -1.49 | 0.138 |
| 12-13 x Tenderness | -3.69 | 2.70 | -1.37 | 0.171 |
| 14-15 x Tenderness | -2.46 | 3.24 | -0.76 | 0.448 |
| 8-9 x Joy | -1.10 | 2.40 | -0.46 | 0.648 |
| 10-11 x Joy | -1.58 | 2.49 | -0.64 | 0.526 |
| 12-13 x Joy | -0.13 | 2.59 | -0.05 | 0.960 |
| 14-15 x Joy | 1.14 | 3.15 | 0.36 | 0.718 |
| 8-9 x Anger | 6.30 | 2.48 | 2.54 | 0.011 |
| 10-11 x Anger | 2.59 | 2.53 | 1.02 | 0.307 |
| 12-13 x Anger | 1.31 | 2.63 | 0.50 | 0.618 |
| 14-15 x Anger | 2.20 | 3.18 | 0.69 | 0.490 |
| 8-9 x Sadness | -8.66 | 2.40 | -3.61 | <0.001 |
| 10-11 x Sadness | -3.71 | 2.49 | -1.49 | 0.137 |
| 12-13 x Sadness | -5.50 | 2.62 | -2.10 | 0.036 |
| 14-15 x Sadness | -5.40 | 3.07 | -1.76 | 0.079 |
| 8-9 x Fear | 6.20 | 2.44 | 2.54 | 0.011 |
| 10-11 x Fear | 3.68 | 2.51 | 1.47 | 0.143 |
| 12-13 x Fear | 5.76 | 2.67 | 2.15 | 0.032 |
| 14-15 x Fear | 0.28 | 3.16 | 0.09 | 0.928 |

**Supplementary Table 28**

*Effects of age group and experienced emotions on HR (ANOVA)*

| Factor | SS | MS | df | F | p |
| --- | --- | --- | --- | --- | --- |
| Age group | 43.40 | 10.85 | 4 | 4.27 | 0.003 |
| Category | 85.50 | 17.10 | 5 | 6.74 | <0.001 |
| Age group x Category | 31.59 | 1.58 | 20 | 0.62 | 0.897 |

**Supplementary Table 29**

*Results of the categorical age model for experienced emotions: HR*

| Predictors | Est/Beta | SE | t | p |
| --- | --- | --- | --- | --- |
| (Intercept) | 77.68 | 1.66 | 46.68 | <0.001 |
| 8-9 | 6.70 | 2.40 | 2.80 | 0.006 |
| 10-11 | 1.52 | 2.50 | 0.61 | 0.543 |
| 12-13 | -0.89 | 2.67 | -0.33 | 0.741 |
| 14-15 | -4.91 | 3.11 | -1.58 | 0.118 |
| Tenderness | -0.13 | 0.27 | -0.51 | 0.613 |
| Joy | 0.52 | 0.27 | 1.97 | 0.049 |
| Anger | -0.28 | 0.28 | -1.01 | 0.314 |
| Sadness | -0.87 | 0.27 | -3.26 | 0.001 |
| Fear | 0.68 | 0.27 | 2.52 | 0.012 |
| 8-9 x Tenderness | -0.43 | 0.40 | -1.06 | 0.288 |
| 10-11 x Tenderness | -0.21 | 0.41 | -0.51 | 0.608 |
| 12-13 x Tenderness | -0.23 | 0.45 | -0.51 | 0.610 |
| 14-15 x Tenderness | 0.20 | 0.53 | 0.37 | 0.709 |
| 8-9 x Joy | -0.06 | 0.38 | -0.16 | 0.870 |
| 10-11 x Joy | 0.41 | 0.40 | 1.02 | 0.306 |
| 12-13 x Joy | -0.36 | 0.43 | -0.84 | 0.400 |
| 14-15 x Joy | -0.16 | 0.52 | -0.31 | 0.755 |
| 8-9 x Anger | 0.37 | 0.40 | 0.93 | 0.351 |
| 10-11 x Anger | -0.12 | 0.41 | -0.30 | 0.766 |
| 12-13 x Anger | 0.44 | 0.43 | 1.01 | 0.314 |
| 14-15 x Anger | 0.41 | 0.52 | 0.79 | 0.432 |
| 8-9 x Sadness | 0.46 | 0.38 | 1.20 | 0.230 |
| 10-11 x Sadness | 0.57 | 0.40 | 1.42 | 0.156 |
| 12-13 x Sadness | 0.76 | 0.43 | 1.78 | 0.075 |
| 14-15 x Sadness | 0.31 | 0.50 | 0.62 | 0.533 |
| 8-9 x Fear | -0.12 | 0.39 | -0.31 | 0.755 |
| 10-11 x Fear | -0.12 | 0.40 | -0.30 | 0.765 |
| 12-13 x Fear | -0.02 | 0.45 | -0.05 | 0.959 |
| 14-15 x Fear | -0.40 | 0.52 | -0.77 | 0.442 |

**Supplementary Table 30**

*Effects of age group and experienced emotions on HRV (ANOVA)*

| Factor | SS | MS | df | F | p |
| --- | --- | --- | --- | --- | --- |
| Age group | 846.27 | 211.57 | 4 | 4.93 | 0.001 |
| Category | 472.10 | 94.42 | 5 | 2.20 | 0.053 |
| Age group x Category | 1362.05 | 68.10 | 20 | 1.59 | 0.051 |

**Supplementary Table 31**

*Results of the categorical age model for experienced emotions: HRV*

| Predictors | Est/Beta | SE | t | p |
| --- | --- | --- | --- | --- |
| (Intercept) | 38.57 | 4.45 | 8.67 | <0.001 |
| 8-9 | 20.65 | 6.40 | 3.22 | 0.002 |
| 10-11 | 25.78 | 6.67 | 3.86 | <0.001 |
| 12-13 | 23.17 | 7.15 | 3.24 | 0.002 |
| 14-15 | 19.96 | 8.33 | 2.40 | 0.018 |
| Tenderness | -0.48 | 1.09 | -0.44 | 0.658 |
| Joy | 0.50 | 1.09 | 0.46 | 0.645 |
| Anger | 0.78 | 1.13 | 0.69 | 0.492 |
| Sadness | -0.01 | 1.09 | -0.01 | 0.993 |
| Fear | -1.23 | 1.11 | -1.11 | 0.267 |
| 8-9 x Tenderness | 1.79 | 1.66 | 1.08 | 0.281 |
| 10-11 x Tenderness | 1.27 | 1.70 | 0.75 | 0.455 |
| 12-13 x Tenderness | -2.52 | 1.85 | -1.36 | 0.174 |
| 14-15 x Tenderness | -0.50 | 2.19 | -0.23 | 0.819 |
| 8-9 x Joy | -2.69 | 1.58 | -1.70 | 0.090 |
| 10-11 x Joy | -1.85 | 1.64 | -1.13 | 0.261 |
| 12-13 x Joy | 0.14 | 1.76 | 0.08 | 0.936 |
| 14-15 x Joy | -2.63 | 2.12 | -1.24 | 0.217 |
| 8-9 x Anger | -2.48 | 1.65 | -1.50 | 0.134 |
| 10-11 x Anger | -0.48 | 1.69 | -0.28 | 0.776 |
| 12-13 x Anger | -1.64 | 1.79 | -0.92 | 0.359 |
| 14-15 x Anger | -0.48 | 2.14 | -0.22 | 0.824 |
| 8-9 x Sadness | 2.96 | 1.58 | 1.87 | 0.062 |
| 10-11 x Sadness | 0.71 | 1.64 | 0.43 | 0.667 |
| 12-13 x Sadness | -0.52 | 1.76 | -0.29 | 0.769 |
| 14-15 x Sadness | 0.27 | 2.06 | 0.13 | 0.897 |
| 8-9 x Fear | 1.88 | 1.61 | 1.17 | 0.243 |
| 10-11 x Fear | 0.59 | 1.66 | 0.36 | 0.722 |
| 12-13 x Fear | 5.65 | 1.83 | 3.09 | 0.002 |
| 14-15 x Fear | 6.48 | 2.13 | 3.05 | 0.002 |

**Supplementary Table 32**

*Effects of age group and experienced emotions on gaze behaviour (ANOVA)*

| Factor | SS | MS | df | F | p |
| --- | --- | --- | --- | --- | --- |
| Age group | 156.09 | 39.02 | 4 | 2.24 | 0.069 |
| Category | 1542.65 | 308.53 | 5 | 17.75 | <0.001 |
| Age group x Category | 430.14 | 21.51 | 20 | 1.24 | 0.218 |

**Supplementary Table 33**

*Categorical age model for experienced emotions: gaze behaviour*

| Predictors | Est/Beta | SE | t | p |
| --- | --- | --- | --- | --- |
| (Intercept) | 89.29 | 1.49 | 60.02 | <0.001 |
| 8-9 | -5.21 | 2.07 | -2.52 | 0.013 |
| 10-11 | -1.11 | 2.17 | -0.51 | 0.608 |
| 12-13 | 0.40 | 2.25 | 0.18 | 0.858 |
| 14-15 | -2.01 | 2.61 | -0.77 | 0.443 |
| Tenderness | -0.28 | 0.73 | -0.38 | 0.704 |
| Joy | 0.66 | 0.73 | 0.90 | 0.371 |
| Anger | 0.55 | 0.75 | 0.73 | 0.464 |
| Sadness | -0.83 | 0.73 | -1.13 | 0.260 |
| Fear | -0.82 | 0.75 | -1.10 | 0.270 |
| 8-9 x Tenderness | 1.92 | 1.07 | 1.79 | 0.074 |
| 10-11 x Tenderness | -0.50 | 1.09 | -0.45 | 0.651 |
| 12-13 x Tenderness | 0.02 | 1.16 | 0.02 | 0.986 |
| 14-15 x Tenderness | 0.45 | 1.37 | 0.33 | 0.741 |
| 8-9 x Joy | 1.90 | 1.02 | 1.85 | 0.064 |
| 10-11 x Joy | 1.61 | 1.07 | 1.51 | 0.132 |
| 12-13 x Joy | 0.90 | 1.11 | 0.81 | 0.419 |
| 14-15 x Joy | 0.04 | 1.33 | 0.03 | 0.974 |
| 8-9 x Anger | -1.37 | 1.06 | -1.29 | 0.197 |
| 10-11 x Anger | 0.85 | 1.08 | 0.79 | 0.429 |
| 12-13 x Anger | 0.75 | 1.12 | 0.67 | 0.506 |
| 14-15 x Anger | 1.06 | 1.34 | 0.79 | 0.427 |
| 8-9 x Sadness | -1.11 | 1.02 | -1.09 | 0.278 |
| 10-11 x Sadness | -1.35 | 1.07 | -1.26 | 0.209 |
| 12-13 x Sadness | -1.29 | 1.13 | -1.15 | 0.252 |
| 14-15 x Sadness | -0.70 | 1.29 | -0.54 | 0.590 |
| 8-9 x Fear | -2.56 | 1.04 | -2.46 | 0.014 |
| 10-11 x Fear | -2.30 | 1.08 | -2.14 | 0.033 |
| 12-13 x Fear | -2.11 | 1.15 | -1.83 | 0.067 |
| 14-15 x Fear | -1.35 | 1.33 | -1.01 | 0.313 |

### Analysis of missing values

Depending on the modality, we only had missing data for between one and seven participants out of our complete sample of 120 participants (Sup. Table 3). We went through all the modalities and considered the potential impact on results, focusing on whether age and emotion category effects could be affected by missing data.

Our additional analyses included calculating descriptive statistics to characterise missing data, as well as chi-square tests and t-tests to test the relationship between emotion category and missing data, and age and missing data, respectively.

The chi-square tests for testing the relationship between emotion category and missing data in all modalities (ratings, HR and HRV, and gaze) were non-significant, suggesting that the missing data were distributed at random across emotion categories. In cases where a participant had missing data, they had it for all emotion categories. Thus, we conclude that missing data likely does not explain our findings on the effect of the emotion category.

For age, we calculated the association between age and missing data using t-tests. The t-tests showed significant results, but they were driven by single missing participants. Because the number of missing data points was low in general, the association between age and missing data became easily significant even when a single participant was missing data. Thus, we investigated each modality and each missing participant separately. Below, we explain in detail the ages of the missing participants and evaluate their effect on the data.

For rating data, we had only one (13-year-old) participant with completely missing data. After removing the missing data, twelve 13-year-old children and 23 12–13-year-old children remained in the data. Also, data were missing equally for all emotion categories, so this did not impact the effect across emotion categories. We conclude that the missing data in ratings are negligible and do not likely affect the results.

For HR and HRV data, we had six participants with completely missing data. For the rest of the participants, we acquired data for all emotion categories. Participants with missing data included one 8-year-old, one 10-year-old, one 12-year-old, two 13-year-olds, and one 15-year-old. Thus, we had complete HR and HRV data from a total of 10 8-year-olds, 10 10-year-olds, 9 12-year-olds, 11 13-year-olds, and 6 15-year-olds. Thus, the missing data were spread evenly across the child age range, which reduces the likelihood of age effects being affected by the missing data. Especially since HR and HRV showed the typical age-related trend even with the missing data, we conclude that missing data do not affect our findings.

For gaze data, we had five participants with completely missing data. Participants with missing data included three adults, one 11-year-old, and one 13-year-old. Thus, most of the missing data were in adults—the missing participants included two 21-year-olds and one 25-year-old. However, we still had 27 adult participants from the age range of 19–29. Our findings showed the main effect of emotion and an interaction effect between age and fear, but no main effect of age. The results presented in Figure 2 show robust trends, and it is unlikely that the amount of missing data (N=6) would affect these trends calculated based on the complete data (N=123).

Thus, after careful consideration of the results together with an investigation of the missing data, we conclude that the data appear to be missing at random and are unlikely to affect our findings.
